# Population Genomics of the Facultatively Mutualistic Bacteria *Sinorhizobium meliloti* and *S. medicae*

**DOI:** 10.1371/journal.pgen.1002868

**Published:** 2012-08-02

**Authors:** Brendan Epstein, Antoine Branca, Joann Mudge, Arvind K. Bharti, Roman Briskine, Andrew D. Farmer, Masayuki Sugawara, Nevin D. Young, Michael J. Sadowsky, Peter Tiffin

**Affiliations:** 1 Department of Plant Biology, University of Minnesota, St. Paul, Minnesota, United States of America; 2 National Center for Genome Resources, Santa Fe, New Mexico, United States of America; 3 Department of Computer Science and Engineering, University of Minnesota, Minneapolis, Minnesota, United States of America; 4 Department of Soil, Water, and Climate, University of Minnesota, St. Paul, Minnesota, United States of America; 5 Department of Plant Pathology, University of Minnesota, Saint Paul, Minnesota, United States of America; 6 BioTechnology Institute, St. Paul, Minnesota, United States of America; Yale University, United States of America

## Abstract

The symbiosis between rhizobial bacteria and legume plants has served as a model for investigating the genetics of nitrogen fixation and the evolution of facultative mutualism. We used deep sequence coverage (>100×) to characterize genomic diversity at the nucleotide level among 12 *Sinorhizobium medicae* and 32 *S. meliloti* strains. Although these species are closely related and share host plants, based on the ratio of shared polymorphisms to fixed differences we found that horizontal gene transfer (HGT) between these species was confined almost exclusively to plasmid genes. Three multi-genic regions that show the strongest evidence of HGT harbor genes directly involved in establishing or maintaining the mutualism with host plants. In both species, nucleotide diversity is 1.5–2.5 times greater on the plasmids than chromosomes. Interestingly, nucleotide diversity in *S. meliloti* but not *S. medicae* is highly structured along the chromosome – with mean diversity (θ_π_) on one half of the chromosome five times greater than mean diversity on the other half. Based on the ratio of plasmid to chromosome diversity, this appears to be due to severely reduced diversity on the chromosome half with less diversity, which is consistent with extensive hitchhiking along with a selective sweep. Frequency-spectrum based tests identified 82 genes with a signature of adaptive evolution in one species or another but none of the genes were identified in both species. Based upon available functional information, several genes identified as targets of selection are likely to alter the symbiosis with the host plant, making them attractive targets for further functional characterization.

## Author Summary

Facultative mutualisms are relationships between two species that can live independently, but derive benefits when living together with their mutualistic partners. The facultative mutualism between rhizobial bacteria and legume plants contributes approximately half of all biologically fixed nitrogen, an essential plant nutrient, and is an important source of nitrogen to both natural and agricultural ecosystems. We resequenced the genomes of 44 strains of two closely related species of the genus *Sinorhizobium* that form facultative mutualisms with the model legme *Medicago truncatula*. These data provide one of the most complete examinations of genomic diversity segregating within microbial species that are not causative agents of human illness. Our analyses reveal that horizontal gene transfer, a common source of new genes in microbial species, disproportionately affects genes with direct roles in the rhizobia-plant symbiosis. Analyses of nucleotide diversity segregating within each species suggests that strong selection, along with genetic hitchhiking has sharply reduced diversity along an entire chromosome half in *S. meliloti*. Despite the two species' ecological similarity, we did not find evidence for selection acting on the same genetic targets. In addition to providing insight into the evolutionary history of rhizobial, this study shows the feasibility and potential power of applying population genomic analyses to microbial species.

## Introduction

Analyses of genome sequences can provide a nearly complete description of the nature and extent of nucleotide diversity segregating within and among species. There have been *multiple investigations into genomic diversity in microbial communities using library-based and* megtagenomic approaches [1] and phylogenomic studies of relatedness among microbial species [2]. By contrast, there have been few genome-wide surveys of nucleotide diversity within a prokaryotic species, and those studies have often focused on variation in genome content [3]–[5] rather than nucleotide diversity. Yet it is clear that population-genomic analyses provide an opportunity to greatly expand our understanding of the evolutionary forces shaping diversity within prokaryotic lineages [6]–[9] and identify targets of strong positive selection without bias that may be introduced when focusing on a limited number of genes or phenotypes of prior interest [10].

Prokaryotic species are often studied because they are either pathogens, of environmental or industrial importance, or because they form mutualistic associations with eukaryotes. The latter group includes members of the genera *Rhizobium*, *Sinorhizobium* (now *Ensifer*), *Bradyrhizobium*, *Azorhizobium*, and *Mesorhizobium*, collectively referred to as the rhizobia, a group of gram-negative bacteria that form symbiotic associations with legume plants. When growing in symbiosis with legumes, rhizobia convert atmospheric nitrogen (N_2_), which is unavailable to plants, into ammonia, which plants can use for the synthesis of amino acids. This symbiosis is estimated to contribute nearly half of all current biological nitrogen fixation [11] and is a key component of agricultural systems that are not dependent on synthetic fertilizers [12].

One of the best characterized rhizobial species is *Sinorhizobium meliloti* (now *Ensifer meliloti)*. The interaction between *S. meliloti* and the closely related species *S. medicae* with the model legume *M. truncatula*, the genome of which was recently sequenced [13], has been the subject of extensive biochemical, molecular genetic [14]–[16], and evolutionary investigation [17]–[20]. The genomes of both S. *meliloti* and *S. medicae* consist of a single circular chromosome (∼3.65 Mb) plus two large symbiotic (sym) plasmids (∼1.3 and ∼1.6 Mb) [21], [22]. *Sinorhizobium* spp. also contain auxiliary plasmids, the number and identity of which varies widely among strains [23] and the functional importance of which is largely unknown. In *Sinorhizobium*, the genes required for forming nodules with legume hosts (including *nod*, *exo*, and *nif* genes) are distributed across both the chromosome and each of the two mega plasmids (hereafter referred to as plasmids) [21], [22], [24]. Bailly et al. [25] recently used low-coverage (∼0.8× average) genomic sequence data to characterize variation in gene content and nucleotide diversity on the chromosomes and two plasmids among 12 *S. medicae* strains. Their coverage was, however, too shallow to robustly characterize nucleotide variation along the genome or search for signatures of recent selection.

In this study we used Illumina technology to sequence the genomes of 12 *S. medicae* and 32 *S. meliloti* strains to over 100× mean depth. We aligned the Illumina data to the *S. meliloti* RM1021 and *S. medicae* WSM419 reference genomes (the chromosome and two plasmids) from each species and then used the aligned sequences to *i)* search for evidence of recent horizontal gene transfer between species, *ii)* characterize genome-wide nucleotide diversity within each species, and *iii*) identify genes that bear the signature of recent positive selection.

## Results

We aligned an average of ∼1,287 Mbp of sequence from each of 12 *S. medicae* and 32 *S. melliloti* strains resulting in median aligned coverage of >100 reads site^−1^ (Tables S1 and S2). For all six replicons (the chromosome and two plasmids of each species) the vast majority of sites were covered by either >50 or <2 reads (Figure S1). The regions with very low coverage are likely either present in the reference genome but not the resequenced strains, are <91% identical in the two strains, and thus too diverged to have aligned using our alignment parameters, or do not align to a single region in the reference genome. Because sequence reads were required to have a single alignment to the reference genome, reads that align to multiple locations were not included in final analyses. In *S. medicae*, an average of 95%, 79%, and 95% of the positions along the reference chromosome, pSMED02, and pSMED01 sequences, respectively, were covered by ≥10 uniquely aligned reads for each resequenced strain (Table S1). In *S. meliloti*, an average of 95%, 71%, and 93% of the positions along the reference chromosome, pSymA, and pSymB, respectively, were covered by ≥10 uniquely aligned reads for each resequenced strain (Table S2. Note, pSMED02 is orthologous to pSymA and pSMED01 is orthologous to pSymB). The high percentage of the reference sequence that can be aligned to the sequence data from the resequenced strains indicate that the vast majority of the sequence found in the reference genomes in each species is also found in all of our resequenced strains.

### Species relatedness

**Figure 1:**
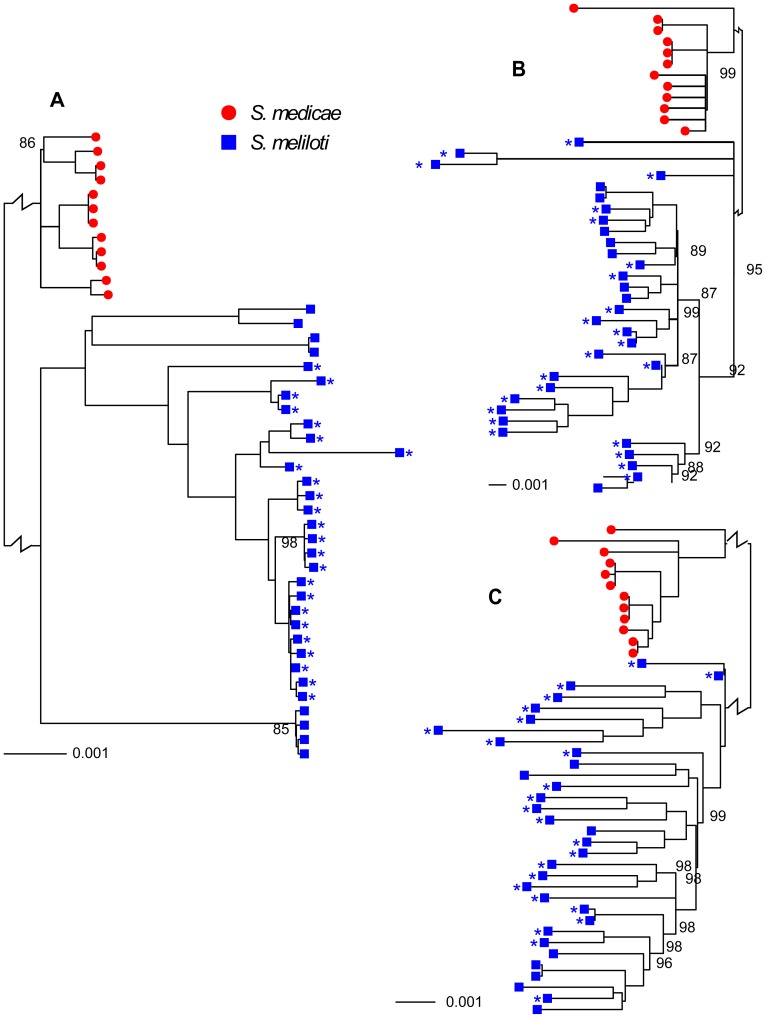
Neighbor-joining trees showing relationships among 32 *S. meliloti* (blue squares) and 12 *S. medicae* (red circles). A) chromosomes, B) pSymA and pSMED02, and C) pSymB and pSMED01. Trees were constructed using sequences from coding regions only. The length of the branch separating *S. medicae* from *S. meliloti* strains is shown at a scale that is 5% of the true scale. The 24-strain *S. meliloti* group is marked by asterisks. All branches had 100% bootstrap support unless otherwise indicated. Branches with <80% bootstrap support were collapsed into polytomies. An identical tree with strain identifications is provided as Figure S2.

*Sinorhizobium medicae* and *S. meliloti* are closely related, have very similar host ranges, and at least partially overlapping geographic ranges [26], [27], characteristics that would provide considerable opportunity for horizontal gene transfer (HGT). Nevertheless, these are clearly distinct species; the chromosomes and plasmids from each species were reciprocally monophyletic (Figure 1) and the number of fixed differences between species greatly exceeded the number of shared polymorphisms (Table 1, Figure S3).

**Table 1:** Number of fixed differences and shared polymorphisms and the ratio of shared polymorphisms to fixed differences between *S. meliloti* and *S. medicae* protein coding genes.

	HGT	Genes	Fixed differences	Shared polymorphisms	Ratio
**Chromosome**	No	2719	273,091	390	0.0015∶ 1
	Yes	1	19	8	0.42∶1
**pSymA/pSMED02**	no	230	25,480	246	0.0010∶ 1
	yes	75	563	1,964	3.49∶ 1
**pSymB/pSMED01**	no	940	107,991	354	0.003∶ 1
	yes	21	383	567	1.48∶ 1

Genes are separated by genomic location and whether they bore a signature of horizontal gene transfer (HGT).

Although we found no evidence for interspecific transfer of whole plasmids, there are 97 genes (1 located on the chromosome, 21 on pSymB/Smed01, and 75 on pSymA/Smed02) with a ratio of shared polymorphisms to fixed differences >0.2, indicative of transferred alleles segregating within the recipient lineage (Table 1, Figure S3). Among these 97 genes (Table S3) are many with clear potential to alter the efficacy of nodulation or nitrogen metabolism including 11 *fix*, 13 *nod*, 8 *nif*, 2 *noe*, 2 *nol*, 5 *rkp* and 3 *syr* genes. By contrast, only 12 *fix*, 7 *nod*, 7 *rkp*, and no *nif*, *noe*, *nol*, or *syr* genes for which the data meet the coverage criteria had a ratio of shared polymorphisms to fixed differences <0.2.

To gain insight into the origin and fate of horizontally transferred genes we clustered the putatively transferred genes into contiguous genomic regions (horizontally transferred genes separated only by genes which did not have a putative ortholog in the reference genome of the other species or by ≤2 genes with ratios of shared polymorphism <0.2) then used neighbor joining trees to examine within and between species relationships. On pSymB/pSMED01, 20 of the 21 putatively transferred genes were found within a single 38 kb region. On pSymA/pSMED02, 6 of the putatively transferred genes are located within a 10.5 kb region of the *S. medicae* reference genome and 62 are located within an ∼300 kb region of the reference genomes. This 300 kb region also contains 236 genes that are present in the *S. medicae* genome (∼102 in *S. meliloti*) for which there was no identifiable ortholog in the reference genome of the other species (Table S3).

**Figure 2:**
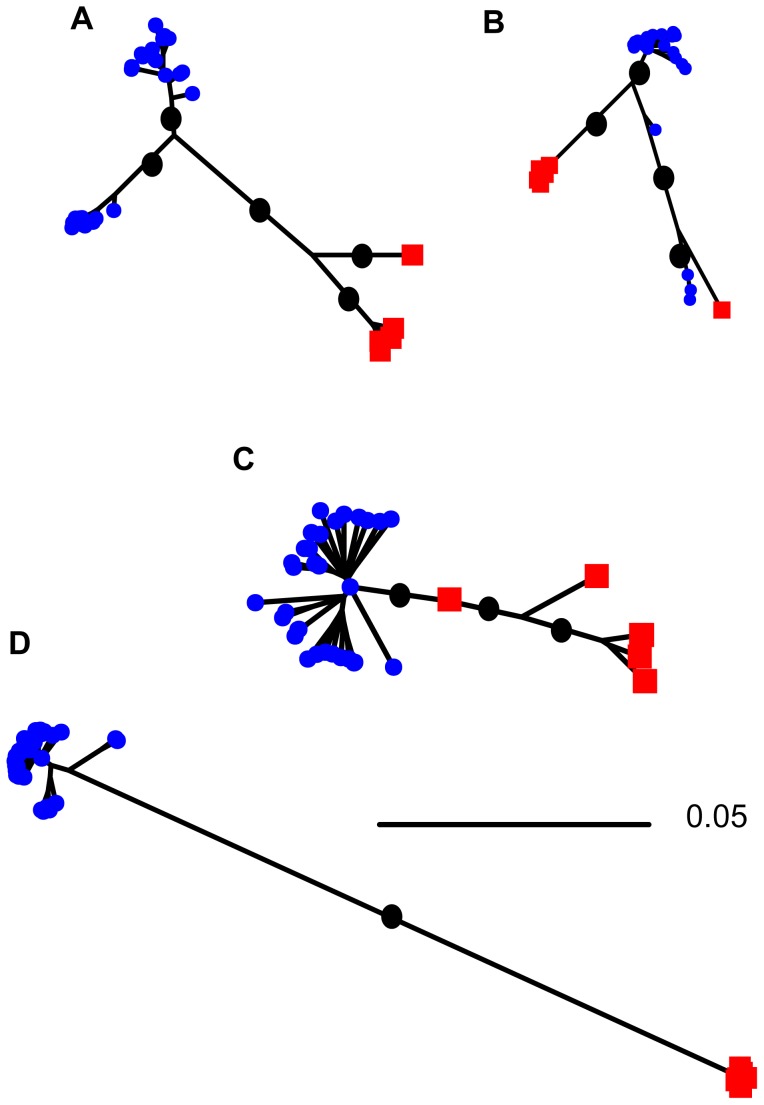
Neighbor-joining trees showing relationships among sequences sampled from *S. meliloti* (blue squares) and *S. medicae* (red circles) for genes showing evidence of horizontal gene transfer. The largest three regions of transferred genes are shown A) 11 genes from pSMED02, concatenated length of 9,291 bp, B) 69 genes from pSMED02, concatenated length of 69,048 bp, C) 23 genes from pSMED01, concatenated 22,493 bp, and D) genes on pSymB for which there was no evidence of horizontal transfer between species (944 genes concatenated 977,757 bp). Black dots indicate major branches with bootstrap support >95%, bootstrap support for shorter branches within single-species clades not shown.

Neighbor joining trees of the large transferred regions (Figure 2), as well as other putatively transferred genes (Figure S4), suggest the history of HGT is complex. For all regions harboring genes with evidence of transfer, the majority of sequences from each species are monophyletic but the branch length separating sequences from the two species is much shorter than the length of the branch separating the two species at genes that show no signal of HGT (Figure 2). There are five regions, all of them on pSymA/pSMED02, for which the putatively transferred genes are not monophyletic (Figure S4); three for which a single *S. medicae*-like sequence was sampled from an *S. meliloti* strain, one for which a *S. meliloti*-like sequence was sampled from an *S. medicae* strain, and one for which the longest branch on the tree separates four sequences (three sampled from *S. meliloti* and one from *S. medicae*) from all other sequences. Interestingly, for this latter case, these four sequences were all sampled from Syria, suggesting geographic structuring of horizontal gene transfer and symbiotic gene alleles.

### Within-species diversity

**Figure 3:**
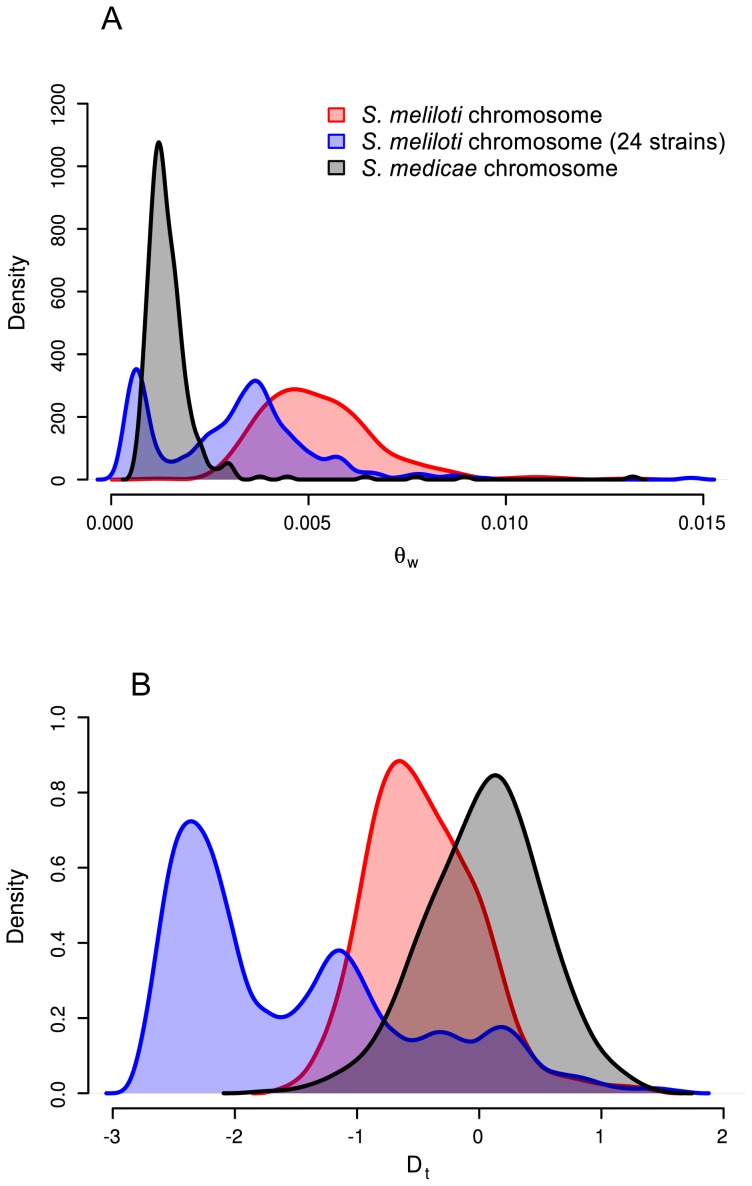
Distributions of chromosomal nucleotide diversity statistics. A) θ_W_ and B) *D_T_* calculated on non-overlapping 10,000 base pair sliding windows. Only windows for which >8,000 bp were covered in >80% of strains are included. Plots were drawn using the R density function with the cosine smoothing kernel [60].

Consistent with previous multi-locus sequence [17], [20], [28] and genomic hybridization data [29] we found 1.5–2 times greater nucleotide diversity on each of the *S. medicae* plasmids than on the *S. medicae* chromosome (Table 2). For the *S. medicae* chromosome, Tajima's D (*D_T_*) was unimodal and centered near zero (Figure 3), a distribution consistent with a panmictic, neutrally evolving population (*i.e.* the standard neutral model [SNM]). Diversity within the full 32 strain sample of *S. meliloti* (Table 2) was two to three times greater than diversity within *S. medicae* but showed the same broad pattern of higher diversity on the plasmids than the chromosome (Table 2). The distribution of chromosomal *D_T_* values in the 32 strain *S. meliloti* sample was negatively centered (Figure 3) and the frequency spectrum of chromosomal polymorphisms revealed a mode of minor alleles found in four strains (Figure S5). This pattern is not consistent with a sample drawn from a single panmictic population and the chromosomal genealogy shows that the 32 strain sample was comprised of three distinct clades; one 24 strain clade and two 4-strain clades (Figure 1).

**Table 2:** Total number of SNPs, nucleotide diversity (mean θ_π_) and mean *D_T_* for all data and for gene regions separated by species and replicon.

	Global			Genes			
	SNPs	θ_π_	*D_t_*	SNPs	θ_π_	θ_π_ syn	*D_t_*
***S. medicae***							
Chromosome	19,547	0.0019	0.014	13,865	0.0016	0.0042	0.04
pSMED02	16,178	0.0056	−0.56	16,178	0.0045	0.0105	−0.14
pSMED01	17,853	0.0031	−1.07	17,853	0.0027	0.0070	−1.04
***S. meliloti***							
Chromosome	74,037	0.0048	−0.43	59,390	0.0045	0.0135	−0.38
pSymA	50,553	0.0126	−0.91	32,619	0.0116	0.0308	−0.86
pSymB	56,398	0.0078	−0.57	41,590	0.0072	0.0193	−0.89
***S. meliloti*** ** 24 **							
Chromosome	39,618	0.0022	−1.20	32,185	0.0020	0.0059	−1.11
First half	26,953	0.004	−0.66	22,042	0.0034	0.010	−0.63
Second half	12,665	0.0008	−2.23	10,143	0.0008	0.002	−1.68
pSymA	46,192	0.0134	−0.73	29,617	0.0123	0.0380	−0.74
pSymB	51,829	0.0079	−0.49	39,221	0.0075	0.0200	−0.51

Data are only from genes for which >90% of the sites have unambiguous base calls from ≥80% of the strains and were not identified as recently transferred between *S. meliloti* and *S. medicae*.

**Figure 4:**
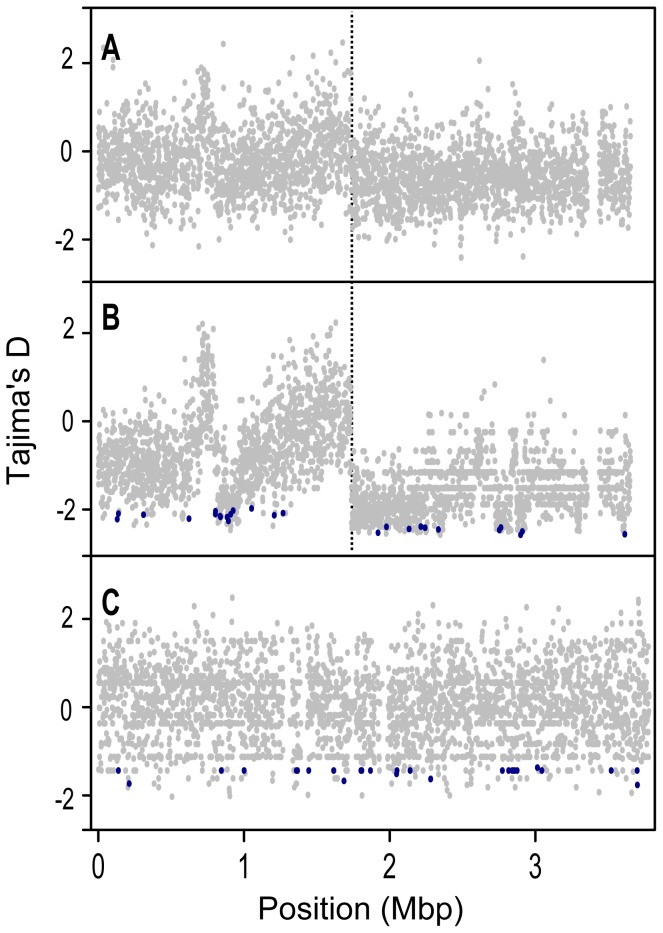
Tajima's D (*D_T_*) values for protein coding genes along the length of the chromosome. A) all 32 *S. meliloti* strains, B) the *S. meliloti* 24 strain group, and C) *S. medicae*. Genes identified by the *D_T_H* test as targets of recent selection are shown in dark blue. Chromosomes are represented linearly, using the coordinate system of the respective reference genomes, with 0 on the far left (and far right); orthologous homologous positions in the two species are not aligned to each other. The origin of replication in *S. meliloti* is at position 0 [61], and the putative location of the terminus is marked with a dotted vertical line.

To remove confounding effects that population structure can have on nucleotide diversity, we recalculated diversity statistics using the sample of 24 *S. meliloti* strains that comprised the largest subpopulation found in our 32 strain sample (Figure 1). Unlike the 32 strain sample, for which the distribution of θ_W_ was unimodal, the distribution of θ_W_ values from the 24 strain sample was distinctly bimodal – with a considerable portion of genes having very low diversity (Figure 3). Similarly, the distribution of *D_T_* values from the 24 strain sample was multi-modal and both far more widely dispersed and more negative than the distribution in the 32 strain sample. This strongly skewed distribution appears to be largely due to genes located on the second half of the chromosome (bp 1,735,000–3,654,135); genes on this half have both low θ_W_ and low *D_T_* values (Figure 4). At both sides of this region, near the origin and terminus of replication there are sharp increases in the per-gene θ_W_ and *D_T_* values. Moreover, neither the first half of the chromosome (Figure S6) nor the plasmids show the well defined 24-strain clade seen in the whole-chromosome genealogy (Figure 1). Taken together, the lack of congruence between genealogies constructed from the two chromosome halves and plasmids as well as the sharp breaks in patterns of diversity provide evidence for transfer of large parts of the plasmids and chromosome among strains of *S. meliloti*.

### Targets of selection

To identify targets of recent adaptation we used the joint *D_T_H* statistic that provides a relatively powerful test of selection that is robust against demographic effects [30]. In *S. medicae*, 27 chromosomal, 9 pSMED01, and 4 pSMED02 genes were identified as putative targets of recent selection. Because the 32 strain sample of *S. meliloti* was strongly affected by population structure we searched for targets of selection in only the 24 strain sample. Moreover, because diversity in this sample was strongly structured along the length of the chromosome, we applied the *D_T_H* test to the first and second halves of the chromosome separately. These analyses identified 15 and 11 genes on the first and second halves of the chromosome, respectively, 11 pSymB, and 5 pSymA genes that harbored signatures of selection (Table S4). None of the genes identified as targets of selection were identified in both species, although *fts* genes, which are annotated as being involved in cell division and are down-regulated in bacteroids [31] were identified as targets of selection in both species (*ftsW* in *S. medicae*, *ftsZ1* and *ftsZ2* in *S. meliloti*). Consistent with the lack of between species overlap in the genes that harbor signatures of recent selection, between-species correlations in nucleotide diversity (θ_W_, *D_T_*) were low for each of the three replicons (all R<0.26). Such low correlations are unexpected if selective constraints or among-gene variance in mutation rates are important determinants of nucleotide diversity and are similar in the two species.

The genes identified as putative targets of selection have a variety of annotated functions. Some of these functions are related to survival or reproduction either inside of nodules or in the soil environment, i.e. osmotic tolerance and stress (*gst9*, *cysK2*, *guaB*, *hutH2*, *oxyR*) and nutrient acquisition (*phoU*, *thuR*). Other putatively selected genes have functions that may be directly related to symbiosis, including *hemA* which is essential for symbiotic nitrogen fixation in many rhizobia, *glgC* and *rkpJ* which affect exopolysaccharide biosynthesis or export which is required that is essential for nodulation, as well as *ftsW*, *ftsZ1*, *ftsZ2*.

## Discussion

Rhizobia species are important symbionts of legume plants and this symbiosis is responsible for approximately half of all current biologically fixed nitrogen [11]. Because of this importance the biochemical and genetic basis of the symbiosis has been subject to extensive investigation [14]–[16]. To gain insight into genomic diversity segregating within rhizobial species, we sequenced to high coverage the genomes of 32 strains of *S. meliloti* and 12 strains of *S. medicae*, the two primary rhizobia symbionts of the model legume *Medicago truncatula*. Our analyses provide insight into the genome-scale extent of horizontal gene transfer (HGT), the structuring of nucleotide diversity within rhizobial genomes, and identify genes that have been subject to recent adaptive evolution in these species.

Previous analyses of genetic diversity within *Sinorhizobium* and other groups of rhizobia [20], [32]–[34] have found clear evidence that genes directly involved in nodule formation can be transferred between species; indeed, in prokaryotes genes involved in symbiosis are often found on mobile elements [35]. Consistent with these previous analyses, our whole-genome analyses revealed 97 genes, most of which are clustered on just three symplasmid regions, for which *S. medicae* and *S. meliloti* genes had a high ratio of shared to fixed polymorphisms, suggestive of recent horizontal transfer between these species. Genes with potential to alter nodulation or nitrogen fixation are overrepresented among the putatively transferred genes, suggesting that HGT may be important in the evolution of symbiosis. At the same time, the importance of HGT in shaping nucleotide diversity is largely restricted to the plasmids and appears to have very little effect on nucleotide variation in genomic regions outside of nodulation-gene islands.

Neighbor-joining trees constructed from plasmid genes show striking differences between genes that show signatures of HGT and those that do not. For genes that do not show evidence of transfer the branch separating sequences from different species is considerably longer than the branches separating sequences sampled from the same species. By contrast, genes that show evidence of transfer have comparatively short branches separating sequences sampled from the two species and relatively long branches separating sequences sampled from the same species. The short branch separating the sequences sampled from the two species suggests that transfer has occurred relatively recently, followed by the transferred sequence spreading through the recipient species. Interestingly, however, sequences from even these transferred genes are largely monophyletic. The sequence similarity of these transferred regions may facilitate ongoing transfer through homologous recombination [36] – thereby preserving these as islands of HGT. The single chromosomal gene with a high ratio of shared to fixed polymorphisms indicates that HGT of chromosomal genes is possible, even if HGT doesn't have an important effect on chromosomal sequence diversity.

### Nucleotide Diversity

The picture of diversity segregating in *S. meliloti* is highly dependent upon the composition of the sample. In our sample of 32 strains, the distributions of summary statistics (i.e. θ_W_ and *D_T_*) are largely consistent with a panmictic population. However, the frequency spectrum of polymorphic sites and genealogical relationships indicate that the 32 strain sample is composed of several distinct subpopulations with 24 strains forming a single well defined clade. The reasons for this substructure are not clear; the members of the 24 strain group were sampled from a wide geographic area (including France, Jordan, Syria, Tunisia), from the full spectrum of geographic locations that the 32 strains were sampled, and from multiple species of host plant (including *M. truncatula*, *M. rigidula*, and *M. sativa*, Table S2 and Figure S2). Regardless of the causes of the population structure, we found a close correspondence between the three major clades identified by whole-genome sequence data and relationships inferred from a 10 locus MLST characterization of population structure within *S. meliloti* [37], [38]. There were 17 strains in common between the two studies, 15 of these were part of the 24 strain group we identified and were monophyletic in the MLST analysis (13 were members of a single MLST group) and the two additional strains were each members of different MLST clusters. The similarity suggests that MLST data provide robust descriptions of population structure in this species.

Interestingly, patterns of diversity segregating among the 24 strain subpopulation are fundamentally different than those found in the 32 strain sample. Most strikingly, for the 24 strain sample, the two halves of the chromosome harbor distinctly different patterns of diversity, with one half of the chromosome having very low values of *D_T_* and θ_W_ relative to the other half. There are several possible causes for the two halves of the chromosome having such different patterns. One possibility is that recent HGT or balancing selection in the 24 strain sample has led to excess diversity and intermediate frequency variants on the high-diversity half of the chromosome. In fact, a neighbor-joining tree made with data from only the high-diversity half of the chromosome shows that some of the strains included in the 24 strain group cluster with strains that are not included in this group (Figure S6). The alternative possibility is that the low-diversity half of the chromosome has experienced a recent selective sweep, leading to reduced diversity and an excess of low-frequency variants. A recently introgressed region segregating at low frequency or the inclusion of a sequence from a divergent subpopulation could also explain the excess of low-frequency variants (low *D_T_* values) found on the second half of the chromosome. Either of these possibilities would cause elevated nucleotide diversity. By contrast, nucleotide diversity in this chromosomal half is reduced relative to the rest of the genome.

To determine if the data are more consistent with excess diversity on the first half of the chromosome or low diversity on the second half of the chromosome we compared nucleotide diversity on each chromosome half to diversity found segregating on the plasmids. For *S. medicae* the chromosome harbors 33 or 60% of the diversity segregating on pSMED02 and pSMED01, respectively. The ratio of chromosomal to plasmid diversity is similar for the *S. meliloti* chromosome from the 32 strain group and the first half of the chromosome from the 24 strain group, these samples harbor >30% and >50% of the diversity segregating on pSymA and pSymB, respectively. By contrast, the low-diversity, second half of the chromosome in the 24 strain group harbors 6% and 10% of the diversity segregating on pSymA and pSymB respectively. To the extent that plasmid diversity can be used as a reference, these data suggest that the distinctly different patterns of diversity found on the two chromosome halves of the 24-strain group may be due to a recent selective sweep that was strong enough to reduce diversity along the entire 1.8 Mb half of the chromosome through genetic hitchhiking [39].

If a selective sweep is responsible for the low diversity on the second half of the chromosome, the sharp borders near the origin and terminus of replication suggests that recombination at the borders is much higher than recombination along the second half of the chromosome or that selection favored the entire region, perhaps due to epistasis between genes located near the borders of the low-diversity region. If a selective sweep is the reason for the reduced diversity than it indicates that genetic hitchhiking along with selective variants could be an extremely important force shaping diversity within natural populations of prokaryotic species and may contribute to driving the divergence between prokaryotic lineages [40].

### Targets of Selection

We identified 82 genes that bear a signature of recent adaptive evolution. These species have similar geographic ranges, life history, and share a common host plant, and as such they may be expected to experience similar selective forces. Nevertheless, the targets of selection in the two species show almost no overlap – no orthologous genes were identified as targets of selection in both species although *fts* genes, involved in cell division, are identified as targets in both species. The lack of overlap in the targets of selection suggest that these two ecologically similar species either experience very different selective forces or that selection acting similarly at the phenotypic level acts on very different genetic targets.

It is notable that no *fix*, *nif*, *nod*, *nol*, *noe*, or *exo* genes, which mutational screens identified as necessary for nodule establishment and nitrogen fixation [14]–[16], are among the genes we identified as bearing a signature of a recent selective sweep. However, nearly all of the *nif*, *nod*, and approximately one-half of the *fix* genes in the *Sinorhizobium* genome showed evidence of HGT or had no reciprocal best sequence match in the other species. Because these genes had no reciprocal best sequence matches we excluded them from our analyses of selection and therefore, their absence from the list of selected genes does not mean they have not been the subject of recent adaptation.

### Summary

Population genetic analyses of nucleotide diversity segregating within *Sinorhizobium medicae* and *S. meliloti* have provided unprecedented insight into the evolutionary history of these ecologically important facultative symbionts. While previous analyses have detected evidence for horizontal gene transfer between these species, our data reveal that gene transfer is restricted almost exclusively to plasmid genes and that the plasmid regions that show evidence of transfer have less interspecific divergence than other genomic regions. Interestingly, nucleotide variation segregating within a 24-strain subpopulation of *S. meliloti* is highly structured along the chromosome, with one half of the chromosome harboring approximately one-fifth as much diversity as the other. The causes of the difference between the two chromosome halves may be a selective sweep coupled with extensive hitchhiking, if this is correct it would suggest that bouts of strong selection may be important in driving the divergence of bacterial species. Finally, we've identified genes that bear a signature of having evolved in response to recent positive selection. Functional characterization of these genes will provide insight into the selective forces that drive rhizobial adaptation.

## Methods

We used Illumina sequencing technology to sequence the genomes of 32 strains of *S. meliloti* and 12 strains of *S. medicae*. These strains were chosen to capture diversity found within the USDA-ARS Rhizobium Germplasm Collection [38], as representative of different multi-locus genotypes [38], or because they had been recently sampled from natural populations and used in experiments to investigate interactions between *Sinorhizobium* and *M. truncatula* [41]. From each strain, DNA was extracted from culture grown cells using the Wizard Genomic DNA Purification kit (Promega Corp. Madison, WI, USA), with further purification by phenol extraction. DNA was then used to construct Illumina paired end libraries using Illumina's phusion-based library kits following the manufacturer's protocols. Insert sizes averaged 332 nt (range = 245 nt to 443 nt). Four samples were multiplexed per lane and sequenced on Illumina GAIIx machines following the manufacturer's protocols. Samples averaged just over 1 Gb of sequence (range = 724 Mb to 1584 Mb) translating into an average and minimum coverage of 174× and 108×, respectively, of the ∼6.7 Mb genome before aligning reads.

For SNP discovery, reads were aligned to the genome sequence of either *S. meliloti* Rm1021 [21], pSymA megaplasmid [42], pSymB megaplasmid [43] and the accessory plasmids pSmeSM11a [44], pSMeSM11b [45] and pRm1132f [46], or *S. medicae* WSM419 chromosome and plasmids pSMED01, pSMED02 and pSMED03 [22], using GSNAP [47] with a 91% minimum identity using the Alpheus pipeline [48]. For this work, only SNPs discovered in the alignment to the chromosome or the megaplasmids (pSymA, pSymB, pSMED01, pSMED02) were used due to poor coverage of the accessory plasmids. Nucleotide identity at a site was called only if that site was covered by ≥10 but <500 uniquely aligned reads (i.e. reads had maximum identity to only a single genomic location) and the nucleotide was supported by ≥70% of unique reads. Positions that did not meet these criteria were treated as ambiguous (N). Sequence reads are available at SRP009881, and SNP data are available for download at http://medicagohapmap.org/.

To evaluate the accuracy of base calls we PCR amplified and Sanger sequenced 25 loci from 4–6 strains (including 3 *S. meliloti* and 3 *S. medicae*) (Table S5). For the 42,953 bp of sequence for which we had both high-quality Sanger and Illumina data that met our coverage criteria there were 157 variants identified by both Sanger and Illumina, 3 variants identified in Sanger but not Illumina, and no variants identified in Illumina but not Sanger data.

Sequences of protein-coding genes were constructed using the IMG version of the WSM419 annotation downloaded on 1 December 2010 and the Rhizobase version of the Rm1021 annotation downloaded on 19 August 2010. For gene-based analyses requiring an outgroup, Rm1021 and WSM419 genes with ≥80% amino acid similarity across ≥80% of their length that were also bidirectional best hits were identified as homologous using the MaGe phyloprofile tool [49]. Gene sequences from the resequenced strains were aligned prior to analyses using the profile alignment tool in ClustalW [50].

### Statistical analyses

We calculated nucleotide diversity for 10 kb non-overlapping windows and for each gene model for which we had data for >90% of the gene length for ≥80% of the strains. The number of replacement and synonymous sites for each gene within each species were estimated using the polydNdS program of the libsequence “analysis” software package [51]. Tajima's D (*D_T_*) [52], the average number of segregating sites (θ_W_) [53] and the average pairwise number of segregating sites (θ_π_) [54] were all estimated using the compute program in libsequence. Fay and Wu's H (*H*) [55] was estimated using a custom libsequence-based program. Because *D_T_* is not defined for genes that have no polymorphism and the distribution of *D_T_* is highly skewed for genes with a single segregating site, we excluded genes with <2 segregating sites from the analysis. The number of fixed differences between *S. medicae* and *S. meliloti* were calculated on biallelic sites in alignments of orthologs using the sharedPoly program (in libsequence). Summary statistics for each of the annotated genes which met coverage criteria are in Dataset S1.

We used the joint *D_T_H* test [30] to look for genes that have experienced recent selective sweeps, considering genes in the lower 5% tail of the distribution for both *D_T_* and *H* as likely targets of selection. We restricted these tests of selection to genes with unambiguous nucleotide calls for >90% of the length from ≥80% of the strains and for which there was no evidence for horizontal gene transfer. For defining the 5% tails we took the ratio of genes that met the coverage and HGT requirement to the total number of genes. Genes with <2 SNPs or without a value for *H* were excluded.

We identified genes likely to have experienced recent horizontal gene transfer by comparing the ratio of polymorphisms that were shared between species to fixed differences between species. Based on the whole-genome distribution of this ratio (Figure S3) we identified putatively transferred genes as those with a ratio of shared polymorphisms to fixed differences >0.2.

### Genealogical relationships

To characterize the genealogical relationships among strains we constructed genealogies using the Neighbor joining algorithm [56] implemented in the dnadist and neighbor programs in Phylip [57] with the F84 model of DNA evolution [58]. Genealogies were constructed using concatenated gene sequences for the chromosome and each of the plasmids separately (2,741 chromosomal genes, 2,668,564 bp; 408 genes on pSymA/pSmed02, 416,009 bp and 1,049 pSymB/pSmed01 genes, 1,084,937 bp). Statistical support for clades in whole-replicon trees was evaluated using 200 bootstrap replicates. NJ trees for genes bearing a signature of horizontal gene transfer were constructed using similar methods, with statistical supported evaluated using 400 bootstrap replicates.

### Origin and terminus of replication

Several analyses were conducted separately for the first and second halves of the *S. meliloti* chromosome. In these cases, we used position 1,735,000 as the dividing line: this position seemed to correspond to the location of the sharp change in *D_T_* along the chromosome. This is also the location of a change in sign of the GC skew in the reference genome, indicating that the terminus of replication is located near this position [59] (Figure S7). GC skew was calculated using a custom R script [60] on all nucleotide positions in 10 kb sliding windows with a 5 kb step. The origin of replication for *S. meliloti* Rm1021 (the reference strain) has been experimentally determined to be near position 0 [61].

## Supporting Information

Figure S1 Distribution of per site coverage on each of the three main replicons (chromosome and two plasmids) from *S. meliloti* and *S. medicae*. The distributions are cumulative distributions for all sequenced strains.
(PDF)

Figure S2 Identical neighbor-joining trees as those shown in Figure 1 with the strain IDs of the 32 *S. meliloti* and 12 *S. medicae* strains labeled. Shown are trees made from A) chromosomes, B) pSymA and pSMED02, and C) pSymB and pSMED01 sequence. Trees were constructed using sequences from coding regions only. The length of the branch separating *S. medicae* from *S. meliloti* strains is shown at a scale that is 5% of the true scale. The 24-strain *S. meliloti* group is marked by asterisks. All nodes were supported by 100% bootstrap support unless otherwise indicated and nodes with <80% bootstrap support were collapsed into polytomies. Strains included in the MLST study by van Berkum et al. [37], [38] are indicated by numbers in parentheses. The strain marked “S” was not assigned to an MLST group by van Berkum et al., but fell in the same clade as the “1” group strains.
(PDF)

Figure S3 Distribution of the ratio of shared polymorphism: fixed differences among protein coding genes with an ortholog in both species that met our coverage criteria. For clarity, the bin on the far right includes genes with a ratio greater than three. (A) pSymB/pSMED01, (B) pSymB/pSMED01, (C) Chromosomes.
(PDF)

Figure S4 Neighbor-joining trees showing relationships among sequences sampled from *S. meliloti* (blue squares) and *S. medicae* (red circles) for genes showing evidence of horizontal gene transfer. The number of genes and base pairs listed for each tree are the numbers concatenated to create the trees, including only genes with that met the coverage criteria in both species and had evidence of horizontal transfer (ratio of shared polymorphisms to fixed differences >0.2). These trees are made from transferred regions based on gene location in the *S. meliloti* genome, whereas those in Figure 2 were based on location in the *S. medicae* reference genome. A) Region 1 on pSymA/pSmed02 (5 genes, 4416 bp). B) Region 2 on pSymA/pSmed02 (1 gene, 942 bp). C) Region 3 on pSymA/pSmed02 (1 gene, 1026 bp). D) Region 4 on pSymA/pSmed02 (55 genes, 52248 bp). E) Region 5 on pSymA/pSmed02 (3 genes, 3293 bp). F) Region 6 on pSymA/pSmed02 (2 genes, 1383 bp). G) Region 1 on pSymB/pSmed01 (16 genes, 15951 bp). H) Region 2 on pSymB/pSmed01 (1 gene, 876 bp). I) The transferred gene on the chromosome: SMc02396 and Smed_0668 (1 gene, 1038 bp).
(PDF)

Figure S5 Minor allele frequency spectrum for the *S. meliloti* chromosome. The MAF spectrum for the full set of strains (A) shows a mode at 4. Reducing the sample to just the 24 strain lineage brings the MAF spectrum closer to neutral expectations (B).
(PDF)

Figure S6 Separate trees for the two halves of the chromosome. The trees presented are majority-rule consensus trees generated from neighbor joining trees created using 500 bootstraps of the concatenated genes that had a putative ortholog in the other species. A) The first half (position 1 to 1735000; 1359 genes, 1314114 bp). B) The second half of the chromosome (position 1735001 to the end; 1361 genes, 1334737 bp). (Positions are given according tothe *S. meliloti* annotation.) The taxa marked with an asterisk (*) belong to the 24 strain group. Bootstrap values are only shown if they are less than 100%, and nodes with bootstrap values less than 80% were collapsed to polytomies.
(PDF)

Figure S7 GC skew along the *S. meliloti* Rm1021 chromosome in 10 kb windows with a 5 kb step. The vertical dotted line at position 1.735 Mbp is the dividing line between the first and second halves of the chromosome and marks a change in sign of the GC skew statistic, a pattern also noted by Capela et al. 2001 [59].
(PDF)

Table S1 Sampling and sequencing information for *S. medicae* strains, listed in alphabetical order, including country of origin, *Medicago* species from which the strain was collected, and sequence coverage data. The percent of the total reads that were aligned to the reference genome is shown for all alignments and for unique alignments (reads that mapped to only one location in the reference). Because most aligned reads aligned uniquely, the rest of the table presents only statistics for uniquely aligned reads.
(DOCX)

Table S2 Sampling and sequencing information for *S. meliloti* strains listed in alphabetical order.
(DOCX)

Table S3 Number and size of putatively transferred regions. Regions were identified in *S. meliloti*, and the size of the region is based on *S. meliloti*. The column labeled “Genes in tracts…” tallies the number of genes that had strong evidence for horizontal gene transfer - they had a shared polymorphisms: fixed differences ratio >0.2 and the gene had adequate coverage in both species. The next two columns tally the total number of genes in the tracts, including those nested between transferred genes. The final column lists the named genes found in the tracts.
(DOCX)

Table S4 Targets of selection listed in order of the unique tags within replicon and species. Gene tags are from IMG annotation of *S. medicae* WSM419 and the Rhizobase annotation of *S. meliloti* Rm1021, annotation information and gene names come from the Rhizobase annotation of *S. meliloti*.
(DOCX)

Table S5 Fragments used to assess the accuracy of Illumina sequence data. For each fragment the start and approximate length of the usable sequence in the *S. melililoti* reference genome is given (*S. medicae* in parentheses). If strains differed in the length of high-quality sequence, the range of fragment lengths is given. *S. meliloti* strains: KH12g, KH16b, KH46c. *S. medicae* strains: KH36d, KH53a, KH53b. PCR conditions were: Cycle 1: 34 cycles 95°C for 0:30, 55°C 1:00, 72°C 2:00. Cycles 2–4 are the same but with 52, 60, or 50°C annealing temperature.
(DOCX)

Dataset S1 Population genetic statistics and annotation for all *S. meliloti* and *S. medicae* protein coding genes in a tab-delimited text file. The comments at the top of the file (lines beginning with “#”) give details on the columns.
(CSV)
