# Supplementary material for: Population Genomics of the Facultatively Mutualistic Bacteria Sinorhizobium meliloti and S. medicae
Source: PLoS Genet. 2012 Aug 2;8(8):e1002868. doi: 10.1371/journal.pgen.1002868 (PMC3410850; doi:10.1371/journal.pgen.1002868)
Supplement: Figure S1 — Distribution of per site coverage on each of the three main replicons (chromosome and two plasmids) from S. meliloti and S. medicae. The distributions are cumulative distributions for all sequenced strains. (PDF) [file pgen.1002868.s001.pdf]

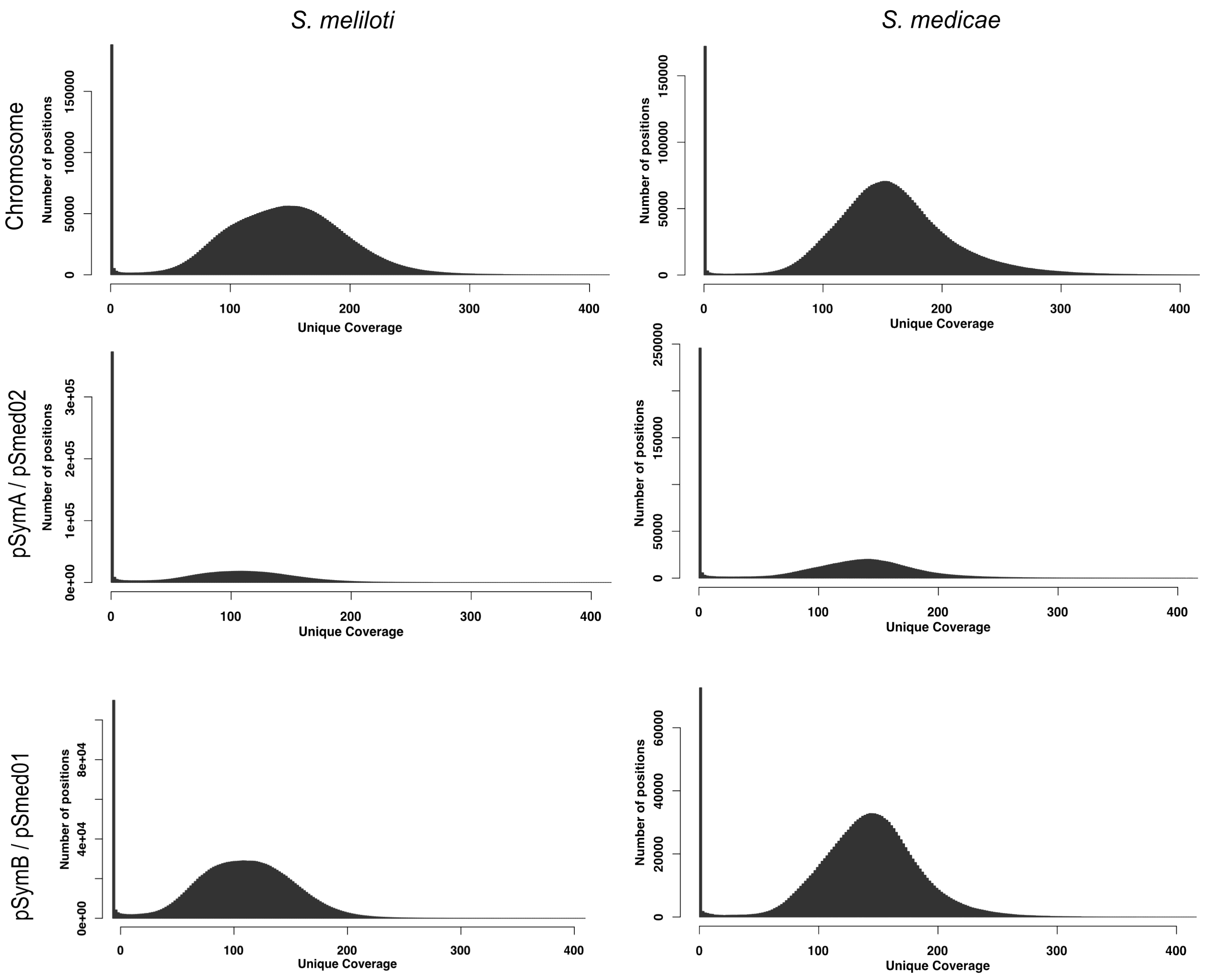

Figure S1: Distribution of per site coverage on each of the three main replicons (chromosome and two plasmids) from *S. meliloti* and *S. medicae*. The distributions are cumulative distributions for all sequenced strains.
