# Supplementary material for: Population Genomics of the Facultatively Mutualistic Bacteria Sinorhizobium meliloti and S. medicae
Source: PLoS Genet. 2012 Aug 2;8(8):e1002868. doi: 10.1371/journal.pgen.1002868 (PMC3410850; doi:10.1371/journal.pgen.1002868)
Supplement: Figure S2 — Identical neighbor-joining trees as those shown in Figure 1 with the strain IDs of the 32 S. meliloti and 12 S. medicae strains labeled. Shown are trees made from A) chromosomes, B) pSymA and pSMED02, and C) pSymB and pSMED01 sequence. Trees were constructed using sequences from coding regions only. The length of the branch separating S. medicae from S. meliloti strains is shown at a scale that is 5% of the true scale. The 24-strain S. meliloti group is marked by asterisks. All nodes were supported by 100% bootstrap support unless otherwise indicated and nodes with <80% bootstrap support were collapsed into polytomies. Strains included in the MLST study by van Berkum et al. [37], [38] are indicated by numbers in parentheses. The strain marked “S” was not assigned to an MLST group by van Berkum et al., but fell in the same clade as the “1” group strains. (PDF) [file pgen.1002868.s002.pdf]

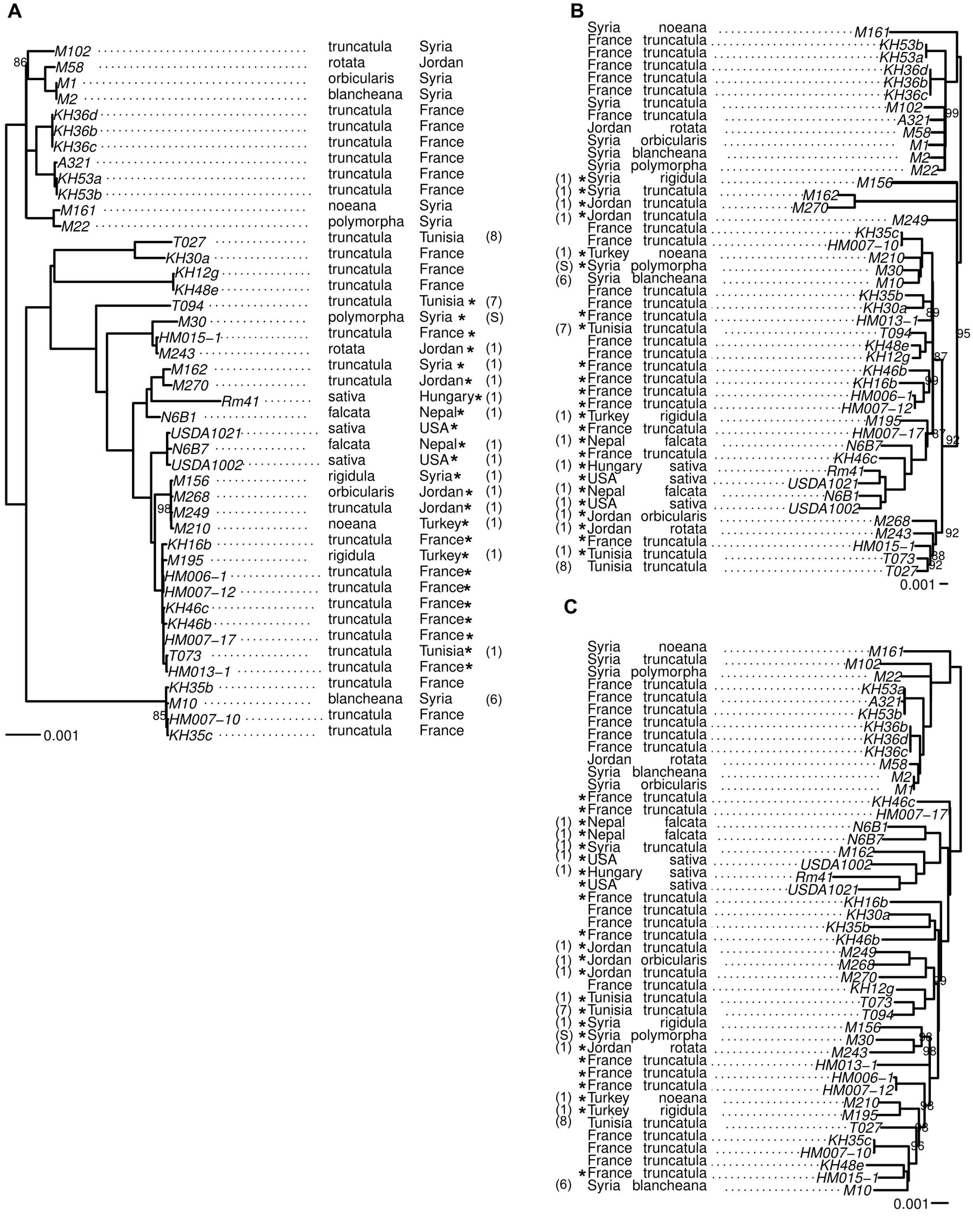

Figure S2: Identical neighbor-joining trees as those shown in Figure 1 with the strain IDs of the 32 *S. meliloti* and 12 *S. medicae* strains labeled. Shown are trees made from A) chromosomes, B) pSymA and pSMED02, and C) pSymB and pSMED01 sequence. Trees were constructed using sequences from coding regions only. The length of the branch separating *S. medicae* from *S. meliloti* strains is shown at a scale that is 5% of the true scale. The 24-strain *S. meliloti* group is marked by asterisks. All nodes were supported by 100% bootstrap support unless otherwise indicated and nodes with < 80% bootstrap support were collapsed into polytomies. Strains included in the MLST study by van Berkum et al. [37,38] are indicated by numbers in parentheses. The strain marked "S" was not assigned to an MLST group by van Berkum et al., but fell in the same clade as the "1" group strains.
