# Supplementary material for: Population Genomics of the Facultatively Mutualistic Bacteria Sinorhizobium meliloti and S. medicae
Source: PLoS Genet. 2012 Aug 2;8(8):e1002868. doi: 10.1371/journal.pgen.1002868 (PMC3410850; doi:10.1371/journal.pgen.1002868)
Supplement: Figure S3 — Distribution of the ratio of shared polymorphism: fixed differences among protein coding genes with an ortholog in both species that met our coverage criteria. For clarity, the bin on the far right includes genes with a ratio greater than three. (A) pSymB/pSMED01, (B) pSymB/pSMED01, (C) Chromosomes. (PDF) [file pgen.1002868.s003.pdf]

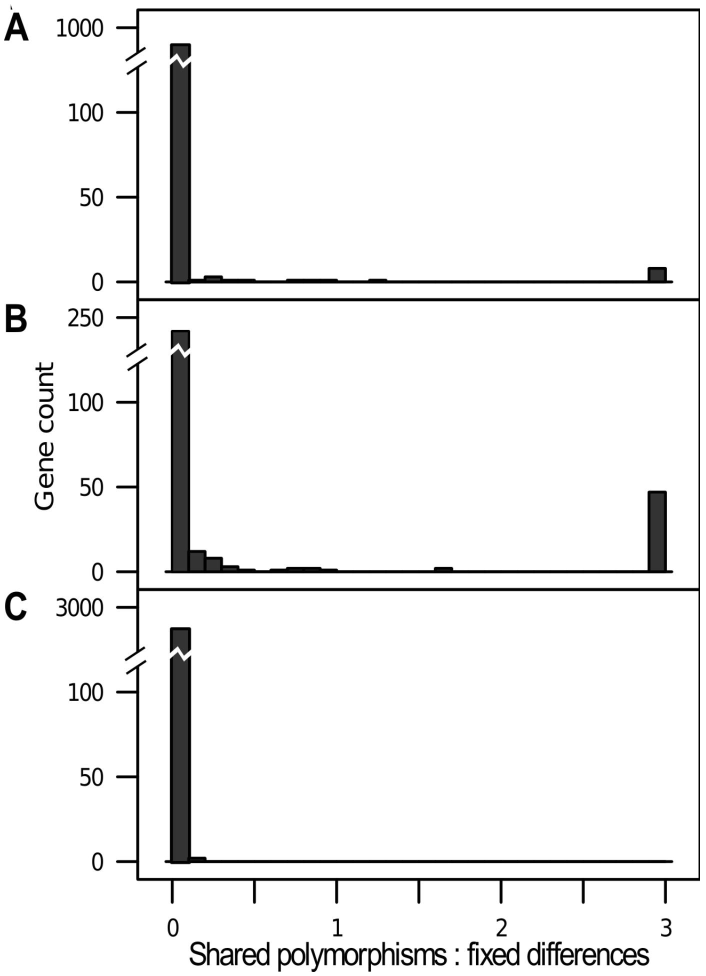

Figure S3: Distribution of the ratio of shared polymorphism: fixed differences among protein coding genes with an ortholog in both species that met our coverage criteria. For clarity, the bin on the far right includes genes with a ratio greater than three. (A) pSymB / pSMED01, (B) pSymB / pSMED01, (C) Chromosomes.
