# Supplementary material for: Population Genomics of the Facultatively Mutualistic Bacteria Sinorhizobium meliloti and S. medicae
Source: PLoS Genet. 2012 Aug 2;8(8):e1002868. doi: 10.1371/journal.pgen.1002868 (PMC3410850; doi:10.1371/journal.pgen.1002868)
Supplement: Figure S4 — Neighbor-joining trees showing relationships among sequences sampled from S. meliloti (blue squares) and S. medicae (red circles) for genes showing evidence of horizontal gene transfer. The number of genes and base pairs listed for each tree are the numbers concatenated to create the trees, including only genes with that met the coverage criteria in both species and had evidence of horizontal transfer (ratio of shared polymorphisms to fixed differences >0.2). These trees are made from transferred regions based on gene location in the S. meliloti genome, whereas those in Figure 2 were based on location in the S. medicae reference genome. A) Region 1 on pSymA/pSmed02 (5 genes, 4416 bp). B) Region 2 on pSymA/pSmed02 (1 gene, 942 bp). C) Region 3 on pSymA/pSmed02 (1 gene, 1026 bp). D) Region 4 on pSymA/pSmed02 (55 genes, 52248 bp). E) Region 5 on pSymA/pSmed02 (3 genes, 3293 bp). F) Region 6 on pSymA/pSmed02 (2 genes, 1383 bp). G) Region 1 on pSymB/pSmed01 (16 genes, 15951 bp). H) Region 2 on pSymB/pSmed01 (1 gene, 876 bp). I) The transferred gene on the chromosome: SMc02396 and Smed_0668 (1 gene, 1038 bp). (PDF) [file pgen.1002868.s004.pdf]

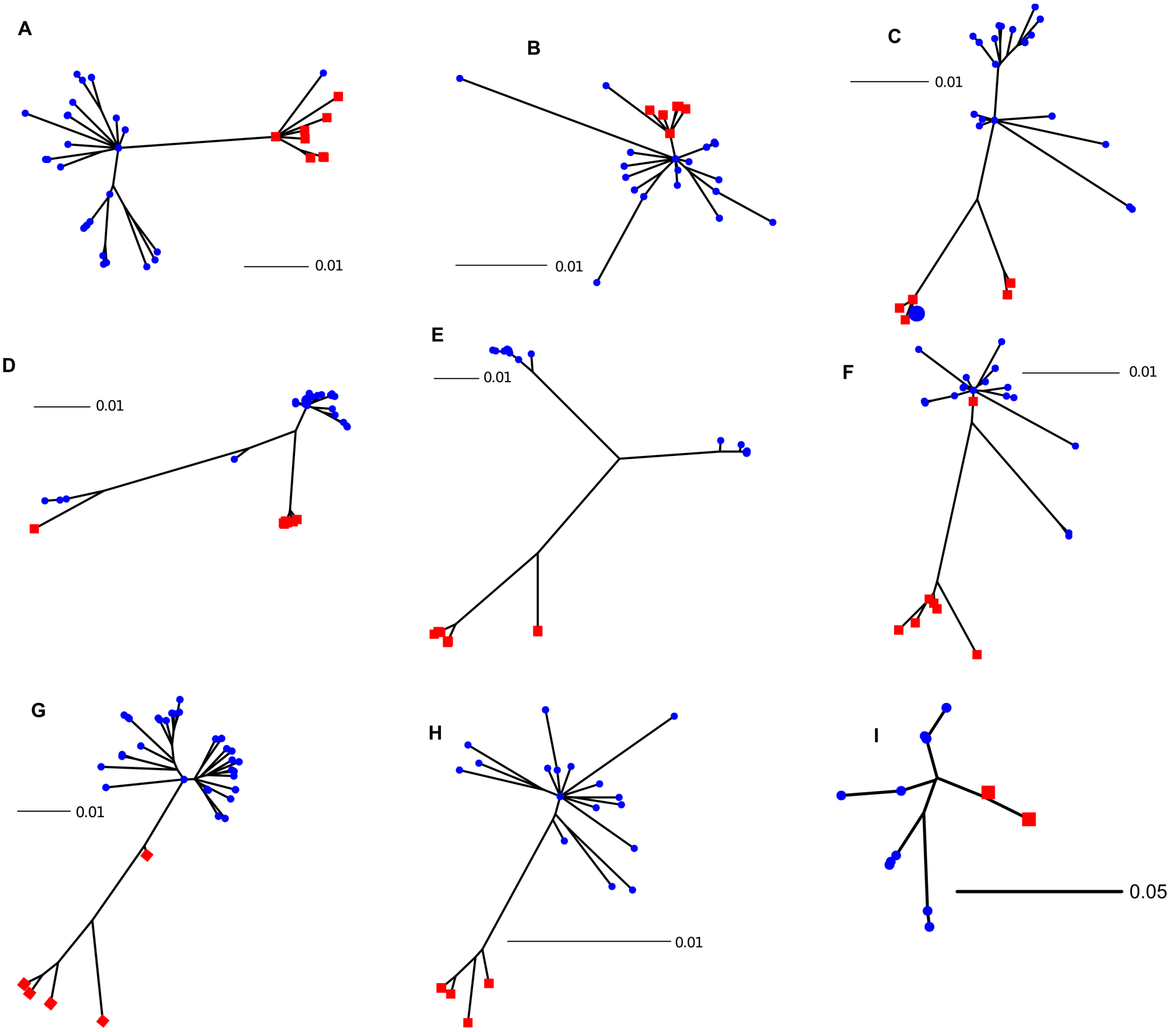


Figure S4. Neighbor joining trees showing relationships among sequences sampled from *S. melitloti* (blue squares) and *S. medicae* (red circles) for genes showing evidence of horizontal gene transfer. The number of genes and base pairs listed for each tree are the numbers concatenated to create the trees, including only genes with that met the coverage criteria in both species and had evidence of horizontal transfer (ratio of shared polymorphisms to fixed differences >0.2). These trees are made from transferred regions based on gene location in the *S. meliloti* genome, whereas those in Figure 2 were based on location in the *S. medicae* reference genome. A) Region 1 on pSymA / pSmed02 (5 genes, 4416 bp). B) Region 2 on pSymA / pSmed02 (1 gene, 942 bp). C) Region 3 on pSymA / pSmed02 (1 gene, 1026 bp). D) Region 4 on pSymA / pSmed02 (55 genes, 52248 bp). E) Region 5 on pSymA / pSmed02 (3 genes, 3293 bp). F) Region 6 on pSymA / pSmed02 (2 genes, 1383 bp). G) Region 1 on pSymB / pSmed01 (16 genes, 15951 bp). H) Region 2 on pSymB / pSmed01 (1 gene, 876 bp). I) The transferred gene on the chromosome: SMc02396 and Smed_0668 (1 gene, 1038 bp).
