# Supplementary material for: Population Genomics of the Facultatively Mutualistic Bacteria Sinorhizobium meliloti and S. medicae
Source: PLoS Genet. 2012 Aug 2;8(8):e1002868. doi: 10.1371/journal.pgen.1002868 (PMC3410850; doi:10.1371/journal.pgen.1002868)
Supplement: Figure S5 — Minor allele frequency spectrum for the S. meliloti chromosome. The MAF spectrum for the full set of strains (A) shows a mode at 4. Reducing the sample to just the 24 strain lineage brings the MAF spectrum closer to neutral expectations (B). (PDF) [file pgen.1002868.s005.pdf]

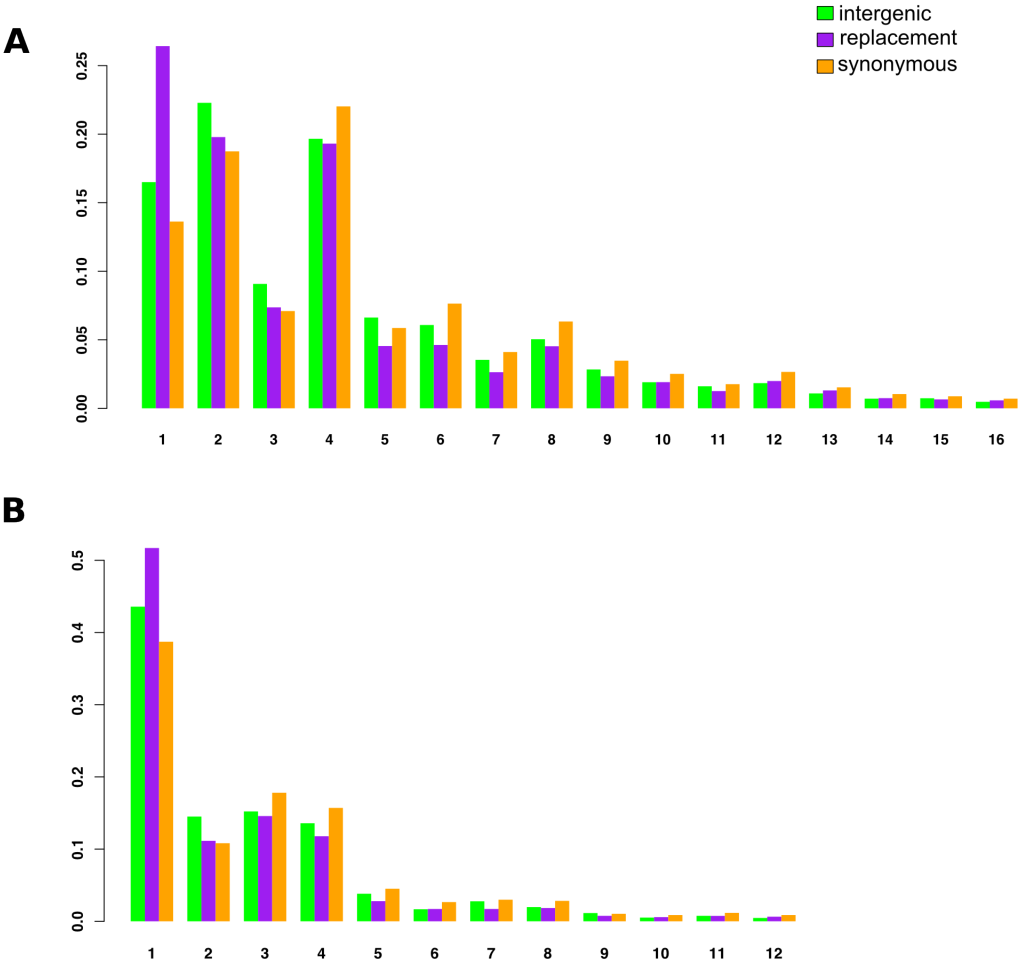

Figure S5: Minor allele frequency spectrum for the *S. meliloti* chromosome. The MAF spectrum for the full set of strains (A) shows a mode at 4. Reducing the sample to just the 24 strain lineage brings the MAF spectrum closer to neutral expectations (B).
