# Supplementary material for: Population Genomics of the Facultatively Mutualistic Bacteria Sinorhizobium meliloti and S. medicae
Source: PLoS Genet. 2012 Aug 2;8(8):e1002868. doi: 10.1371/journal.pgen.1002868 (PMC3410850; doi:10.1371/journal.pgen.1002868)
Supplement: Figure S6 — Separate trees for the two halves of the chromosome. The trees presented are majority-rule consensus trees generated from neighbor joining trees created using 500 bootstraps of the concatenated genes that had a putative ortholog in the other species. A) The first half (position 1 to 1735000; 1359 genes, 1314114 bp). B) The second half of the chromosome (position 1735001 to the end; 1361 genes, 1334737 bp). (Positions are given according tothe S. meliloti annotation.) The taxa marked with an asterisk (*) belong to the 24 strain group. Bootstrap values are only shown if they are less than 100%, and nodes with bootstrap values less than 80% were collapsed to polytomies. (PDF) [file pgen.1002868.s006.pdf]

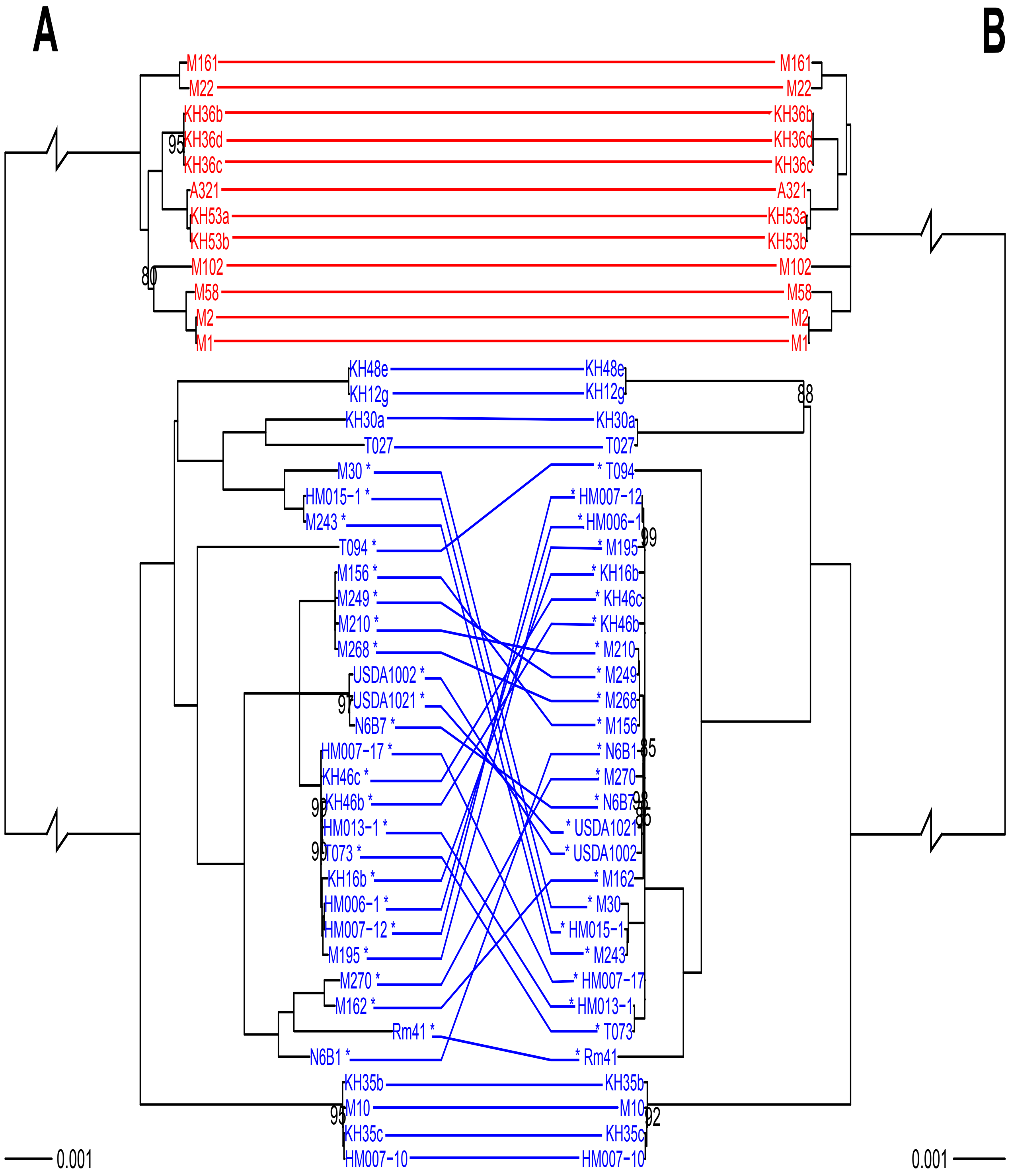

Figure S6. Separate trees for the two halves of the chromosome. The trees presented are majority-rule consensus trees generated from neighbor joining trees created using 500 bootstraps of the concatenated genes that had an ortholog in both species. A) The first half (position 1 to 1735000; 1359 genes, 1314114 bp). B) The second half of the chromosome (position 1735001 to the end; 1361 genes, 1334737 bp). (Positions are given according tothe *S. meliloti* annotation.) The taxa marked with an asterisk (*) belong to the 24 strain group. Bootstrap values are only shown if they are less than 100%, and nodes with bootstrap values less than 80% were collapsed to polytomies.
