# Supplementary material for: Population Genomics of the Facultatively Mutualistic Bacteria Sinorhizobium meliloti and S. medicae
Source: PLoS Genet. 2012 Aug 2;8(8):e1002868. doi: 10.1371/journal.pgen.1002868 (PMC3410850; doi:10.1371/journal.pgen.1002868)
Supplement: Figure S7 — GC skew along the S. meliloti Rm1021 chromosome in 10 kb windows with a 5 kb step. The vertical dotted line at position 1.735 Mbp is the dividing line between the first and second halves of the chromosome and marks a change in sign of the GC skew statistic, a pattern also noted by Capela et al. 2001 [59]. (PDF) [file pgen.1002868.s007.pdf]

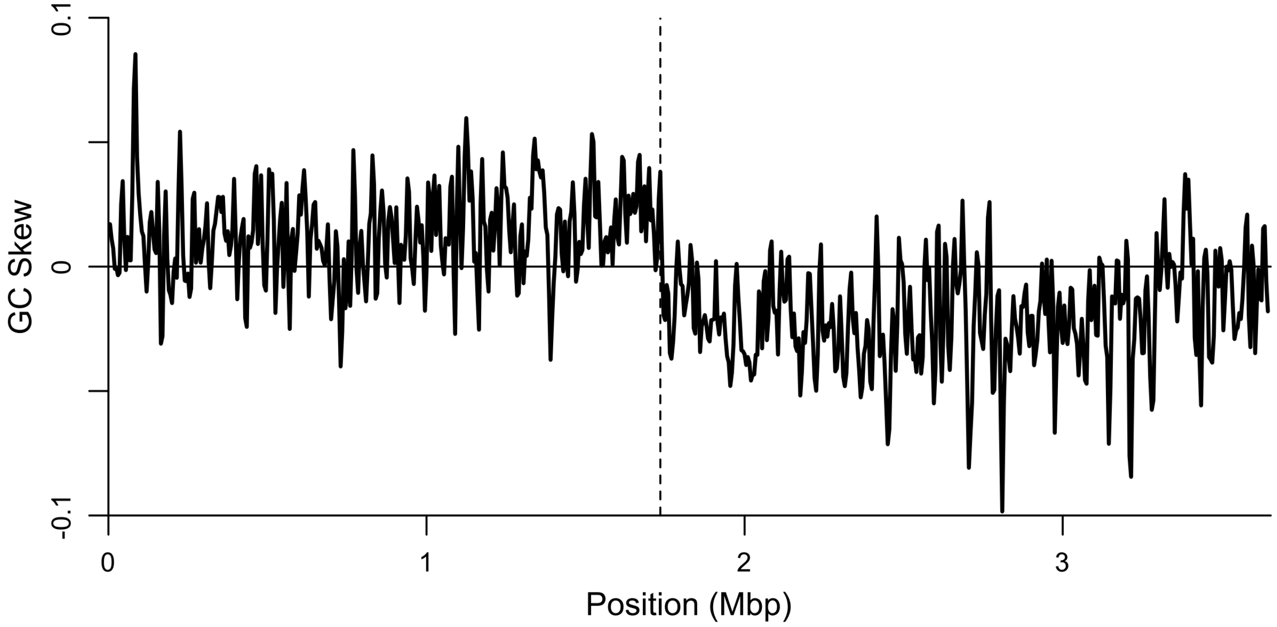

Figure S7. GC skew along the *S. meliloti* Rm1021 chromosome in 10 kb windows with a 5 kb step. The vertical dotted line at position 1.735 Mbp is the dividing line between the left and right halves of the chromosome. Note the change in sign of the GC skew statistic at that position. This pattern was also noted by Capela et al. [59].
