# Supplementary material for: Population Genomics of the Facultatively Mutualistic Bacteria Sinorhizobium meliloti and S. medicae
Source: PLoS Genet. 2012 Aug 2;8(8):e1002868. doi: 10.1371/journal.pgen.1002868 (PMC3410850; doi:10.1371/journal.pgen.1002868)
Supplement: Table S1 — Sampling and sequencing information for S. medicae strains, listed in alphabetical order, including country of origin, Medicago species from which the strain was collected, and sequence coverage data. The percent of the total reads that were aligned to the reference genome is shown for all alignments and for unique alignments (reads that mapped to only one location in the reference). Because most aligned reads aligned uniquely, the rest of the table presents only statistics for uniquely aligned reads. (DOCX) [file pgen.1002868.s008.docx]

Table S1: Information on *S. medicae* strains, listed in alphabetical order, including country of origin, host *Medicago* species from which the strain was collected, and coverage data. The percent of the total number of reads that were aligned to the reference genome is shown for all alignments and for unique alignments (reads that mapped to only one location in the reference). Because most the alignable reads aligned uniquely, the rest of the table presents only statistics for uniquely aligned reads.

|  |  |  |  |  |  |  | Mean unique coverage | | |  | Median unique coverage | | |  | Proportion of sites with ≥ 10 unique reads | | |
| --- | --- | --- | --- | --- | --- | --- | --- | --- | --- | --- | --- | --- | --- | --- | --- | --- | --- |
| Strain | Origin | Host | Total reads ( X 106) | % reads aligned | % reads uniquely aligned |  | Chromosome | pSmed02 | pSmed01 |  | Chromosome | pSmed02 | pSmed01 |  | Chromosome | pSmed02 | pSmed01 |
| A321 | France | *truncatula* | 9.20 | 80.6 | 79.3 |  | 106.6 | 86.1 | 93.3 |  | 109 | 91 | 96 |  | 0.95 | 0.89 | 0.94 |
| KH36b | France | *truncatula* | 12.70 | 83.8 | 82.3 |  | 145.5 | 121.5 | 144.8 |  | 151 | 147 | 150 |  | 0.95 | 0.77 | 0.96 |
| KH36c | France | *truncatula* | 15.88 | 82.5 | 81 |  | 180.6 | 149.1 | 180.1 |  | 185 | 175 | 185 |  | 0.96 | 0.77 | 0.96 |
| KH36d | France | *truncatula* | 8.95 | 88.4 | 87 |  | 110.6 | 86.5 | 109.0 |  | 113 | 99 | 111 |  | 0.95 | 0.77 | 0.96 |
| KH53a | France | *truncatula* | 12.34 | 81.0 | 79.6 |  | 138.2 | 111.5 | 137.7 |  | 141 | 131 | 144 |  | 0.96 | 0.81 | 0.95 |
| KH53b | France | *truncatula* | 12.44 | 87.4 | 86 |  | 152.8 | 119.0 | 147.7 |  | 157 | 142 | 156 |  | 0.96 | 0.79 | 0.94 |
| M1 | Syria | *orbicularis* | 12.97 | 82.8 | 81.2 |  | 147.8 | 121.0 | 142.3 |  | 153 | 137 | 148 |  | 0.94 | 0.84 | 0.95 |
| M102 | Syria | *truncatula* | 14.60 | 83.7 | 82.2 |  | 191.9 | 104.3 | 140.3 |  | 193 | 123 | 144 |  | 0.95 | 0.77 | 0.95 |
| M161 | Syria | *noeana* | 17.61 | 78.9 | 77.6 |  | 215.7 | 120.4 | 166.7 |  | 217 | 147 | 170 |  | 0.96 | 0.70 | 0.95 |
| M2 | Syria | *blancheana* | 12.89 | 82.7 | 81 |  | 146.3 | 125.6 | 142.6 |  | 150 | 140 | 147 |  | 0.94 | 0.84 | 0.95 |
| M22 | Syria | *polymorpha* | 11.90 | 85.1 | 83.8 |  | 153.7 | 103.0 | 118.9 |  | 156 | 113 | 123 |  | 0.96 | 0.86 | 0.95 |
| M58 | Jordan | *rotata* | 12.84 | 82.0 | 80.8 |  | 161.4 | 93.0 | 130.8 |  | 166 | 116 | 135 |  | 0.94 | 0.70 | 0.95 |
|  |  |  |  |  |  |  |  |  |  |  |  |  |  |  |  |  |  |
| Mean | - | - | 12.86 | 83.24 | 81.82 |  | 154.3 | 111.8 | 137.8 |  | 157.6 | 130.1 | 142.4 |  | 0.95 | 0.79 | 0.95 |
