# Supplementary material for: Population Genomics of the Facultatively Mutualistic Bacteria Sinorhizobium meliloti and S. medicae
Source: PLoS Genet. 2012 Aug 2;8(8):e1002868. doi: 10.1371/journal.pgen.1002868 (PMC3410850; doi:10.1371/journal.pgen.1002868)
Supplement: Table S2 — Sampling and sequencing information for S. meliloti strains listed in alphabetical order. (DOCX) [file pgen.1002868.s009.docx]

Table S2: Information on *S. meliloti* strains listed in alphabetical order.

|  |  |  |  |  |  |  |  | Mean unique coverage | | |  | Median unique coverage | | |  | Proportion of sites with ≥ 10 unique reads | | |
| --- | --- | --- | --- | --- | --- | --- | --- | --- | --- | --- | --- | --- | --- | --- | --- | --- | --- | --- |
| Strain | 24 strain group | Origin | Host | Total reads ( X 10^6) | % reads aligned | % reads uniquely aligned |  | Chromosome | pSymA | pSymB |  | Chromosome | pSymA | pSymB |  | Chromosome | pSymA | pSymB |
| HM006-1 | * | France | *truncatula* | 9.3 | 79.7 | 78.8 |  | 107 | 73.8 | 95.4 |  | 106 | 76 | 95 |  | 0.95 | 0.70 | 0.92 |
| HM007-10 |  | France | *truncatula* | 11.9 | 79.2 | 78.3 |  | 138 | 88.7 | 124.6 |  | 147 | 114 | 132 |  | 0.93 | 0.67 | 0.94 |
| HM007-12 | * | France | *truncatula* | 37.4 | 85.7 | 84.7 |  | 96 | 57.3 | 76.6 |  | 98 | 67 | 80 |  | 0.95 | 0.80 | 0.95 |
| HM007-17 | * | France | *truncatula* | 24.2 | 85.7 | 84.7 |  | 85 | 53.4 | 67.5 |  | 86 | 56 | 68 |  | 0.95 | 0.84 | 0.95 |
| HM013-1 | * | France | *truncatula* | 13.5 | 83.8 | 82.8 |  | 173 | 102.7 | 139.5 |  | 179 | 127 | 149 |  | 0.95 | 0.74 | 0.92 |
| HM015-1 | * | France | *truncatula* | 12.1 | 81.7 | 80.8 |  | 157 | 77.0 | 119.0 |  | 163 | 99 | 123 |  | 0.95 | 0.67 | 0.96 |
| KH12g |  | France | *truncatula* | 14.2 | 79.6 | 78.9 |  | 175 | 90.1 | 132.5 |  | 183 | 112 | 142 |  | 0.94 | 0.72 | 0.92 |
| KH16b | * | France | *truncatula* | 14.0 | 77.4 | 76.7 |  | 162 | 91.0 | 130.1 |  | 167 | 107 | 137 |  | 0.95 | 0.76 | 0.93 |
| KH30a |  | France | *truncatula* | 9.6 | 80.2 | 79.5 |  | 119 | 66.6 | 92.4 |  | 125 | 84 | 98 |  | 0.93 | 0.70 | 0.92 |
| KH35b |  | France | *truncatula* | 13.4 | 79 | 78.3 |  | 149 | 117.1 | 141.5 |  | 159 | 147 | 153 |  | 0.93 | 0.73 | 0.91 |
| KH35c |  | France | *truncatula* | 10.8 | 71.9 | 71.2 |  | 109 | 61.6 | 96.1 |  | 114 | 75 | 100 |  | 0.93 | 0.66 | 0.93 |
| KH46b | * | France | *truncatula* | 8.0 | 90 | 89.1 |  | 114 | 61.0 | 85.2 |  | 119 | 75 | 91 |  | 0.95 | 0.71 | 0.91 |
| KH46c | * | France | *truncatula* | 14.4 | 76.4 | 75.4 |  | 164 | 104.2 | 128.4 |  | 170 | 123 | 134 |  | 0.95 | 0.79 | 0.93 |
| KH48e |  | France | *truncatula* | 8.6 | 74.1 | 73.5 |  | 92 | 53.2 | 77.1 |  | 96 | 64 | 79 |  | 0.93 | 0.74 | 0.95 |
| M10 |  | Syria | *blancheana* | 10.3 | 72.6 | 71.7 |  | 121 | 56.9 | 87.5 |  | 127 | 71 | 92 |  | 0.93 | 0.66 | 0.93 |
| M156 | * | Syria | *rigidula* | 12.5 | 78.6 | 77.7 |  | 161 | 66.0 | 118.8 |  | 165 | 75 | 126 |  | 0.95 | 0.58 | 0.92 |
| M162 | * | Syria | *truncatula* | 13.7 | 80.2 | 79.3 |  | 176 | 72.8 | 137.6 |  | 182 | 86 | 146 |  | 0.95 | 0.65 | 0.92 |
| M195 | * | Turkey | *rigidula* | 12.9 | 83.5 | 82.8 |  | 163 | 101.6 | 132.4 |  | 166 | 119 | 135 |  | 0.95 | 0.77 | 0.96 |
| M210 | * | Turkey | *noeana* | 12.8 | 84.2 | 83.1 |  | 169 | 83.2 | 134.1 |  | 174 | 110 | 141 |  | 0.95 | 0.66 | 0.95 |
| M243 | * | Jordan | *rotata* | 11.5 | 79.6 | 78.8 |  | 143 | 76.0 | 113.9 |  | 148 | 97 | 122 |  | 0.95 | 0.66 | 0.91 |
| M249 | * | Jordan | *truncatula* | 12.3 | 79.3 | 78.4 |  | 152 | 79.1 | 121.5 |  | 156 | 101 | 129 |  | 0.95 | 0.63 | 0.93 |
| M268 | * | Jordan | *orbicularis* | 12.9 | 79.9 | 79 |  | 157 | 87.5 | 128.4 |  | 161 | 106 | 135 |  | 0.95 | 0.66 | 0.93 |
| M270 | * | Jordan | *truncatula* | 11.0 | 74.5 | 73.3 |  | 130 | 63.7 | 95.9 |  | 135 | 80 | 102 |  | 0.95 | 0.65 | 0.92 |
| M30 | * | Syria | *polymorpha* | 12.5 | 77.8 | 77 |  | 152 | 82.6 | 116.3 |  | 156 | 101 | 121 |  | 0.95 | 0.70 | 0.94 |
| N6B1 | * | Nepal | *falcata* | 14.0 | 78.1 | 76.9 |  | 169 | 100.7 | 126.6 |  | 174 | 121 | 132 |  | 0.95 | 0.74 | 0.94 |
| N6B7 | * | Nepal | *falcata* | 13.4 | 80 | 78.7 |  | 164 | 103.8 | 125.1 |  | 169 | 116 | 131 |  | 0.95 | 0.85 | 0.94 |
| Rm41 | * | Hungary | *sativa* | 9.0 | 73.6 | 72.8 |  | 96 | 77.2 | 80.7 |  | 99 | 90 | 86 |  | 0.94 | 0.79 | 0.91 |
| T027 |  | Tunisia | *truncatula* | 13.0 | 81.9 | 81.2 |  | 175 | 74.8 | 121.2 |  | 179 | 86 | 126 |  | 0.94 | 0.64 | 0.92 |
| T073 | * | Tunisia | *truncatula* | 10.1 | 77 | 76.1 |  | 126 | 57.6 | 92.9 |  | 125 | 66 | 94 |  | 0.95 | 0.63 | 0.93 |
| T094 | * | Tunisia | *truncatula* | 8.7 | 75.7 | 74.9 |  | 105 | 54.4 | 76.7 |  | 106 | 63 | 79 |  | 0.94 | 0.71 | 0.92 |
| USDA1002 | * | USA | *ativa* | 9.6 | 79.8 | 78.6 |  | 124 | 64.1 | 81.4 |  | 118 | 65 | 78 |  | 0.95 | 0.74 | 0.91 |
| USDA1021 | * | USA | *ativa* | 15.0 | 79.5 | 78.4 |  | 172 | 129.8 | 149.6 |  | 176 | 155 | 159 |  | 0.95 | 0.78 | 0.92 |
|  |  |  |  |  |  |  |  |  |  |  |  |  |  |  |  |  |  |  |
| Mean |  | - | - | 13.0 | 79.4 | 78.5 |  | 140 | 79.0 | 110.8 |  | 145 | 94.8 | 116 |  | 0.95 | 0.71 | 0.93 |
