# Supplementary material for: Population Genomics of the Facultatively Mutualistic Bacteria Sinorhizobium meliloti and S. medicae
Source: PLoS Genet. 2012 Aug 2;8(8):e1002868. doi: 10.1371/journal.pgen.1002868 (PMC3410850; doi:10.1371/journal.pgen.1002868)
Supplement: Table S3 — Number and size of putatively transferred regions. Regions were identified in S. meliloti, and the size of the region is based on S. meliloti. The column labeled “Genes in tracts…” tallies the number of genes that had strong evidence for horizontal gene transfer - they had a shared polymorphisms: fixed differences ratio >0.2 and the gene had adequate coverage in both species. The next two columns tally the total number of genes in the tracts, including those nested between transferred genes. The final column lists the named genes found in the tracts. (DOCX) [file pgen.1002868.s010.docx]

Table S3: Number and approximate size of putatively transferred regions. The regions were identified in *S. meliloti*, and the size of the tract is given for *S. meliloti*. Regions defined as contiguous sets of horizontally transferred genes separated only by genes which did not have a putative ortholog in the reference genome of the other species or by ≤ 2 genes with ratios of shared polymorphism < 0.2he column labeled "Genes in tracts..." tallies the number of genes that had strong evidence for horizontal gene transfer - they had a shared polymorphisms : fixed differences ratio greater than 0.2 and the gene had adequate coverage in both species. The next two columns tally the total number of genes in the tracts, including those nested between transferred genes. The final column lists the named genes found in the tracts, in the order they are located in the reference genome.

| Regions | Size (in kb) | Genes in tracts with shared: fixed > 0.2 | *S. medicae*  total genes in tract | *S. meliloti* total genes in tract | Named genes |
| --- | --- | --- | --- | --- | --- |
| **pSymA / pSmed02** | | |  |  |  |
| 1 | 26 | 5 | 11 | 30 | *fifixP3 fixI2 fixS2 aqpZ2 fixK* |
| 3 | 1 | 1 | 1 | 1 |  |
| 4 | 300 | 62 | 164 | 298 | *arcB arcC dgoK2 cspA6 groEL2 groES2 nodD2 fixT2 fixK2 fixN2 fixO2 fixQ2 fixP2 nodL noeA noeB gabD4 syrB3 fixU fdxN nifB nifA fixX fixC fixB fixA nifH nifD nifK nifE nifX fdxB*  *orf10.5 syrA nodD3 syrM nodH nodF nodE nodG nodP1 nodQ1 nodJ nodI nodC nodB nodA nodD1 orf110 nifN nodN nolG nolF nodM dnaE3 traG traD traC traA1 atrA atrB atrC fabG ntrR2 actP hmrR cyaE2 nex18 tspO cycB1 cyaP hspC2 degP4 cycB2 nosR nosZ nosD nosF nosY nosL nosX fhp nnrS fixS1 fixI1 fixH fixG fixP1* |
| 5 | 1 | 1 | 1 | 1 | *fixJ* |
| 6 | 4 | 4 | 6 | 6 | *napC napB napA napD napG* |
| 7 | 2 | 2 | 2 | 2 | *msrA3* |
|  | | |  |  |  |
| **pSymB / pSmed01** | | |  |  |  |
| 1 | 23 | 20 | 23 | 19 | *kdsB2 kpsF1 msbA1 mocD mocE rkpT2 kpsF2 rkpR/kpsE rkpS rkpT1 rkpZ1* |
| 2 | 2 | 2 | 2 | 2 |  |
