# Supplementary material for: Population Genomics of the Facultatively Mutualistic Bacteria Sinorhizobium meliloti and S. medicae
Source: PLoS Genet. 2012 Aug 2;8(8):e1002868. doi: 10.1371/journal.pgen.1002868 (PMC3410850; doi:10.1371/journal.pgen.1002868)
Supplement: Table S4 — Targets of selection listed in order of the unique tags within replicon and species. Gene tags are from IMG annotation of S. medicae WSM419 and the Rhizobase annotation of S. meliloti Rm1021, annotation information and gene names come from the Rhizobase annotation of S. meliloti. (DOCX) [file pgen.1002868.s011.docx]

Table S4: Targets of selection listed in order of the unique tags within replicon and species. Gene tags are from IMG annotation of *S. medicae* WSM419 and the Rhizobase annotation of *S. meliloti* Rm1021, annotation information and gene names come from the Rhizobase annotation of *S. meliloti*.

| **Gene tag** | ***S. meliloti* annotation** | **Potential role in symbiosis** | **Gene name** |
| --- | --- | --- | --- |
| ***S. medicae* chromosome** | |  |  |
| Smed_0123 | phosphate transporter PhoU | mutations suppress the symbiotic and phosphate-dependent phenotypes associated with mutations in the phoCDET locus [1]. | *phoU* |
| Smed_0193 | capsular polysaccharide biosynthesis/export | KBP production/export, in S. fredii mutants show reduced nodulation on soybean but not cowpea [2] | *rkpJ* |
| Smed_0787 | putative signal peptide |  |  |
| Smed_0925 | putative 2-component receiver domain | cellulose synthesis, which is induced upon contact with roots during attachment [29] and biofilm formation [3]. | *celR1* |
| Smed_1265 | putative peptidyl-prolyl cis-trans isomerase A signal | Down-regulated in hfq mutant: hfq is essential for nodulation and N-fixation [4]. | *ppiA* |
| Smed_1266 | putative peptidyl-prolyl cis-trans isomerase B | Same as *ppiA* | *ppiB* |
| Smed_1274 | putative oxidoreductase |  |  |
| Smed_1363 | cysteine synthase A | Other cys genes (cysG) has delayed nodulation and white nodules [5]; *cysK1* downregulated in *hfq* mutants. | *cysK2* |
| Smed_1528 | hypothetical |  |  |
| Smed_1588 | putative amino-acid binding periplasmic |  |  |
| Smed_1600 | putative sensor histidine kinase transmembrane |  |  |
| Smed_1723 | hypothetical |  |  |
| Smed_1728 | putative periplasmic binding ABC transporter signal pep. |  |  |
| Smed_1785 | hypothetical |  |  |
| Smed_1994 | hypothetical |  |  |
| Smed_1997 | RNA polymerase sigma factor | Differentially expressed by *Sinorhizobium* exposed to *M. sativa* compared to *M. truncatula* roots [6]. | *rpoE1* |
| Smed_2083 | cell division FtsW peptidoglycan synthesis | Down regulated in bacteroids [7]; required for cytokineseis | *ftsW* |
| Smed_2200 | hypothetical |  |  |
| Smed_2205 | putative transcription regulator |  |  |
| Smed_2668 | hypothetical |  |  |
| Smed_2708 | putative membrane-bound lytic murein transglycosylase |  |  |
| Smed_2730 | putative RNA polymerase sigma-E factor (sigma-24) | Gene regulation in response to oxidative, saline, and osmotic stress; upregulated when in contact with *M. sativa* or *M. truncatula* [6]. | *rpoE4* |
| Smed_2741 | glucose-1-phosphate adenylyltransferase | Affects exopolysacharides (glycogen production).G*lgA* mutant in *R. tropici* affects nodulation.[3,8] | *glgC* |
| Smed_2756 | putative adenylate/guanylate cyclase |  | *cyaG1* |
| Smed_2880 | 5-aminolevulinate synthase | Mutants (defective in ALA synthase) able to elicit nodules but nodules unable to fix nitrogen [9,10]. | *hemA* |
| Smed_2901 | putative oxidoreductase |  |  |
| Smed_3323 | putative glutathione S-transferase | expression affects nitrogenase activity and antioxidant defenses ([11]). Glutathione has fundamental role in symbiosis capacity ([12]). | *gst9* |
| Smed_3491, 3493 | hypothetical |  |  |
| ***S. medicae* pSMED01** | |  |  |
| Smed_3766 | putative oxidoreductase |  |  |
| Smed_3813 | putative ThuR regulatory  for trehalosemaltose transport | Alanine metabolism important in pea nodules [13]. | *thuR* |
| Smed_3819 | putative sugar ABC transporter permease |  |  |
| Smed_3821 | putative ABC transporter periplasmic sugar-binding |  |  |
| Smed_3864 | putative D-amino acid dehydrogenase |  |  |
| Smed_3922 | Transketolase |  | *cbbT* |
| Smed_4152 | bifunctional aldehyde dehydrogenase |  | *paaZ* |
| Smed_4155 | putative 3-hydroxyacyl-CoA dehydrogenase |  |  |
| Smed_4620 | xanthine dehydrogenase |  | *xdhA1* |
| ***S. medicae* pSMED02** | |  |  |
| Smed_5148 | transmembrane-transport protein |  |  |
| Smed_5275 | AdeC4 adenine deaminase | ureide formation in soybean nodules [14]. | *adeC4* |
| Smed_5328 | ABC transporter ATP-binding |  |  |
| Smed_5361 | sensor |  |  |
| ***S. meliloti* chromosome** | |  |  |
| SMc00037 | putative transcription regulator |  |  |
| SMc00185 | ABC transporter ATP-binding transmembrane | one of twelve genes upregulated during infection with either *M. sativa* or *M. truncatula* compared to no-plant controls [6]. |  |
| SMc00355 | hypothetical |  |  |
| SMc00489 | hypothetical |  |  |
| SMc00595 | nucleoside diphosphate kinase |  | *ndk* |
| SMc00669 | putative histidine ammonia-lyase | osmoregulation and symbiosis in *S. meliloti* [15]. | *hutH2* |
| SMc00677 | hypothetical |  |  |
| SMc00782 | hypothetical |  |  |
| SMc00783 | hypothetical |  |  |
| SMc00815 | inositol-5-monophosphate dehydrogenase | thermal sensitivity; mutants form nodules but have no rhizobia [16]. | *guaB* |
| SMc00818 | putative hydrogen peroxide-inducible genes activator | Hydrogen peroxide produced inside of nodules during N fixation: *oxyR* mutants more sensitive to H_2_O_2_ [17]; regulates *katA* [18]. | *oxyR* |
| SMc00861 | putative signal peptide |  |  |
| SMc00878 | putative transcription regulator |  |  |
| SMc00898 | glutathione-regulated potassium-efflux system |  | *kefB1* |
| SMc00932 | DNA mismatch repair |  | *mutL* |
| SMc01874 | cell division FtsZ | Down regulated in bacteroids [7]. | *ftsZ1* |
| SMc02257 | putative transport system permease ABC transporter |  |  |
| SMc02334 | putative pentose kinase transmembrane |  |  |
| SMc02342 | transketolase |  | *tkt1* |
| SMc02553 | hypothetical |  |  |
| SMc04134 | putative transcription regulator |  |  |
| SMc04142 | hypothetical |  |  |
| SMc04296 | cell division FtsZ | Overexpression causes altered cell morphology similar to branched and filamentous form found in bacteroids [19]. | *ftsZ2* |
| SMc04315 | putative transcription regulator |  |  |
| SMc04407 | putative transport transmembrane |  |  |
| ***S. meliloti* pSymA** | |  |  |
| SMa0166 | hypothetical protein |  |  |
| SMa1597 | Pilus assembly chaperone |  |  |
| SMa2325 | transcriptional regulator |  |  |
| SMa2349 | putative xanthine dehydrogenase iron-sulfur-binding |  |  |
| SMa2355 | DNA polymerase IV |  |  |
| ***S. meliloti* pSymB** | |  |  |
| SMb20255 | hypothetical |  |  |
| SMb20699 | protein secretion |  |  |
| SMb21155 | hypothetical |  |  |
| SMb21164 | putative formiminoglutamase |  | *hutG* |
| SMb21284 | uricase |  |  |
| SMb21292 | membrane |  |  |
| SMb21324 | putative glucose-1-phosphate thymidyltransferase |  | *wgaG* |
| SMb21378 | hypothetical |  |  |
| SMb21534 | putative dehydrogenase |  |  |
| SMb21536 | hypothetical |  |  |
| SMb21586 | glutathione synthetase | downregulated in *hfq* mutants [4]; glutathione has fundamental role in symbiosis [20]. | *gshB2* |

Table S4 References:

1. Newman JD, Diebold RJ, Schultz BW, Noel KD (1994) Infection of soybean and pea nodules by *Rhizobium spp*. purine auxotrophs in the presence of 5-aminoimidazole-4-carboxamide riboside. J Bacteriol 176: 3286.

2. Hidalgo A, Margaret I, Crespo-Rivas JC, Parada M, Murdoch PS, et al. (2010) The *rkpU* gene of *Sinorhizobium fredii* HH103 is required for bacterial KPS production and for efficient nodulation with soybean but not with Vigna unguiculata. Microbiology 156: 3398–3411.

3. Williams A, Wilkinson A, Krehenbrink M, Russo DM, Zorreguieta A, et al. (2008) Glucomannan-mediated attachment of *Rhizobium leguminosarum* to pea root hairs is required for competitive nodule infection. J Bacteriol 190: 4706 –4715.

4. Barra-Bily L, Fontenelle C, Jan G, Flechard M, Trautwetter A, et al. (2010) Proteomic alterations explain phenotypic changes in *Sinorhizobium meliloti* lacking the RNA chaperone hfq. J Bacteriol 192: 1719 –1729.

5. Pobigaylo N, Szymczak S, Nattkemper TW, Becker A (2008) Identification of genes relevant to symbiosis and competitiveness in *Sinorhizobium meliloti* using signature-tagged mutants. Mol Plant Microbe Interact 21: 219–231.

6. Terpolilli J (2009) Why are the symbioses between some genotypes of Sinorhizobium and Medicago suboptimal for N2 fixation? [Thesis]. Available:http://researchrepository.murdoch.edu.au/683/. Accessed 19 December 2011.

7. Barnett MJ, Toman CJ, Fisher RF, Long SR (2004) A dual-genome symbiosis chip for coordinate study of signal exchange and development in a prokaryote–host interaction. Proc Natl Acad Sci USA 101: 16636 –16641.

8. Ausmees N, Jonsson H, Höglund S, Ljunggren H, Lindberg M (1999) Structural and putative regulatory genes involved in cellulose synthesis in *Rhizobium leguminosarum bv. trifolii*. Microbiology 145: 1253 –1262.

9. Bruijn FJ, Felix G, Grunenberg B, Hoffmann HJ, Metz B, et al. (1989) Regulation of plant genes specifically induced in nitrogen-fixing nodules: role of cis-acting elements and trans-acting factors in leghemoglobin gene expression. Plant Mol Biol 13: 319–325.

10. Stanley J, Dowling DN, Broughton WJ (1988) Cloning of *hemA* from *Rhizobium sp.* NGR234 and symbiotic phenotype of a gene-directed mutant in diverse legume genera. Mol Gen Genet 215: 32–37.

11. Stiens M, Schneiker S, Keller M, Kuhn S, Pühler A, et al. (2006) Sequence analysis of the 144-kilobase accessory plasmid pSmeSM11a, isolated from a dominant *Sinorhizobium meliloti* strain identified during a long-term field release experiment. Appl Environ Microbiol 72: 3662 –3672.

12. Stiens M, Schneiker S, Pühler A, Schlüter A (2007) Sequence analysis of the 181‐kb accessory plasmid pSmeSM11b, isolated from a dominant *Sinorhizobium meliloti* strain identified during a long‐term field release experiment. FEMS Microbiology Letters 271: 297–309.

13. Lodwig E, Kumar S, Allaway D, Bourdes A, Prell J, et al. (2004) Regulation of l-alanine dehydrogenase in *Rhizobium leguminosarum bv. viciae* and its role in pea nodules. J Bacteriol 186: 842 –849.

14. Woo KC, Atkins CA, Pate JS (1980) Biosynthesis of ureides from purines in a cell-free system from nodule extracts of cowpea [*Vigna unguiculata* (L) Walp.]. Plant Physiol 66: 735–739.

15. Boncompagni E, Dupont L, Mignot T, Østeräs M, Lambert A, et al. (2000) Characterization of a *Sinorhizobium meliloti* ATP-Binding cassette histidine transporter also involved in betaine and proline uptake. J Bacteriol 182: 3717 –3725.

16. Riccillo PM, Collavino MM, Grasso DH, England R, de Bruijn FJ, et al. (2000) A *guaB* mutant strain of *Rhizobium tropici* CIAT899 pleiotropically defective in thermal tolerance and symbiosis. Mol Plant Microbe Interact 13: 1228–1236.

17. Meier VM, Muschler P, Scharf BE (2007) Functional analysis of nine putative chemoreceptor proteins in *Sinorhizobium meliloti*. J Bacteriol 189: 1816 –1826.

18. Jamet A, Kiss E, Batut J, Puppo A, Hérouart D (2005) The *katA* catalase gene is regulated by *oxyR* in both free-living and symbiotic *Sinorhizobium meliloti*. J Bacteriol 187: 376 –381.

19. Gibson KE, Kobayashi H, Walker GC (2008) Molecular determinants of a symbiotic chronic infection. Annu Rev Genet 42: 413–441.

20. Harrison J, Jamet A, Muglia CI, Van de Sype G, Aguilar OM, et al. (2005) Glutathione plays a fundamental role in growth and symbiotic capacity of *Sinorhizobium meliloti*. J Bacteriol 187: 168 –174.
