# Supplementary material for: Population Genomics of the Facultatively Mutualistic Bacteria Sinorhizobium meliloti and S. medicae
Source: PLoS Genet. 2012 Aug 2;8(8):e1002868. doi: 10.1371/journal.pgen.1002868 (PMC3410850; doi:10.1371/journal.pgen.1002868)
Supplement: Table S5 — Fragments used to assess the accuracy of Illumina sequence data. For each fragment the start and approximate length of the usable sequence in the S. melililoti reference genome is given (S. medicae in parentheses). If strains differed in the length of high-quality sequence, the range of fragment lengths is given. S. meliloti strains: KH12g, KH16b, KH46c. S. medicae strains: KH36d, KH53a, KH53b. PCR conditions were: Cycle 1: 34 cycles 95°C for 0:30, 55°C 1:00, 72°C 2:00. Cycles 2–4 are the same but with 52, 60, or 50°C annealing temperature. (DOCX) [file pgen.1002868.s012.docx]

Table S5. Fragments used to assess the accuracy of Illumina sequence data. For each fragment the start and approximate length of the usable sequence in the *S. melililoti* reference genome is given (*S. medicae* in parentheses). If strains differed in the length of high-quality sequence, the range of fragment lengths is given. *S. meliloti* strains: KH12g, KH16b, KH46c. *S. medicae* strains: KH36d, KH53a, KH53b. PCR conditions were: Cycle 1: 34 cycles 95 ºC for 0:30, 55 ºC 1:00, 72 ºC 2:00. Cycles 2 – 4 are the same but with 52, 60, or 50 ºC annealing temperature.

| Fragment | Replicon | Primers | PCR cycle | Start | Length | Strains |
| --- | --- | --- | --- | --- | --- | --- |
| asd | Chrom | F: CGGCCGGGAGATGCTGAACA  R: ATGCGCTTGGTGAACTTCTTG | 1 | 3617425 (3347795) | 401 | KH12g KH16b KH36d KH46c KH53a KH53b |
| edD | Chrom | F: GGCATCATCACCTCCTACAA  R: CGGCGTGCCGGGATT | 1 | 767751 (318657) | 441 | KH12g KH16b KH36d KH46c KH53a KH53b |
| gap | Chrom | F: CGGTCCGGTCGAGACCAA  R: CGGTAGAGATCCTTGTGCAT | 1 | 2975612 (2755361) | 376 | KH12g KH16b KH36d KH46c KH53a KH53b |
| glnD | Chrom | F: GTGCGCTGCCACATGCAYTT  R: CCGGRTCRCGCTTGAA | 1 | 32205 (32205) | 216 | KH12g KH16b KH36d KH46c KH53a KH53b |
| gnd | Chrom | F: GGGCCGGCTCAACTCCTA  R: CGGCATCGGCAGGTT | 1 | 2091418 | 288 | KH12g, KH16b, KH46c |
| nodD1 | pSymA / pSMED02 | F: TTCAGGGTTCTCTAATAGG  R: AAGTTAAKGCTCTTGGC | 4 | (1105981) | 903 | KH36d, KH53a, KH53b |
| nodD3 | pSymA / pSMED02 | F: TTCGCCTTTACTGATTGGTCGG  R: ATCCGTCAATGTCTAACGC | 1 | 462810 (1132988) | 882 | KH16b, KH46c, KH53a, KH53b |
| nodH | pSymA / pSMED02 | F: TAGAAGCACGAAACTAGCAAA CG  R: AAGCTCAAAGAACCTCGCG | 1 | 468267 (1119739) | 676 | KH12g, KH16b, KH36d, KH46c, KH53b |
| nodP2 | pSymB / pSMED01 | F: ATTGCGATATGTCTCTTCCC  R: ATCGACTTGTTGTCGTGC | 2 | 786682 (1046202) | 399 – 899 | KH12g KH16b KH36d KH46c KH53a KH53b |
| nolR | Chrom | F: TCTTCGCGAACGATTCCTTCGC  R: TCATGGCGCGATCATGTAGCACG | 3 | 2594603 | 829 | KH12g, KH16b, KH46c |
| nuoE1 | Chrom | F: GCGCGCKCAGGAGCAGGA  R: CGCAGGCGCCCTGACATT | 1 | 1381488 (963622) | 204 | KH12g KH16b KH36d KH46c KH53a KH53b |
| ordL2 | Chrom | F: GCGGCGCGGTCGTCAT  R: CGCCATGGCCGGAATA | 1 | 771164 (322085) | 337 | KH12g KH16b KH36d KH46c KH53a KH53b |
| recA | Chrom | F: CCGGTTCGCTCGGCCTCGATA  R: CGCCCATCTCGCCCTCGATTT | 1 | 1948820 (1607544) | 229 | KH12g KH16b KH36d KH46c KH53a KH53b |
| sucA | Chrom | F: GCTCGGCCTCGAATA  R: CCGTCAGCGACAGGT | 1 | 3315057 (3089072) | 432 | KH12g KH16b KH36d KH46c KH53a KH53b |
| syrM | pSymA / pSMED02 | F: ATATTGTGACGACCTGG  R: ATTCATTATGCTCACCCATCC | 1 | 466411 (1126442) | 977 – 986 | KH16b, KH36d, KH53a |
| val_a4 | pSymA | F: AAAGCAGCCGATATCCAGAA  AAGCCGCGAATATCGAAGTA | 2 | 989078 | 578 – 591 | KH12g, KH16b, KH46c |
| val_b1 | pSymB | F: AGCTGTCTTTCGGCGTAATG  R: GCCATGACCTCCTTCGAT | 1 | 937793 | 481 – 570 | KH12g, KH16b, KH46c |
| val_b2 | pSymB | F: CATCAATCCTTCGCTGTCCT  R: TGGAAA ACTTGTGCAGAATGAC | 2 | 342840 | 557 – 586 | KH12g, KH16b |
| val_b3 | pSymB | F: AAGGAACATCTGGGCCTGC  R: TTTCCTCGGCGCTTTCG | 1 | 1635399 | 404 - 531 | KH12g, KH16b, KH46c |
| val_b4 | pSymB | F: ATA AGGCTGCCGAGGTTCTT  R: ATGATGACCCCAGGATTGC | 1 | 633180 | 429 – 455 | KH16b, KH46c |
| val_b5 | pSymB | F: ATTGCCGGAGAAGTTCATGT  R: AGGAGCCGATGGTTTTCAAG | 2 | 1677556 | 392 | KH16b |
| val_p01_1 | pSMED01 | F: GAGAGGTCGCACTCGTCAAC  R: TGCTGACTGTCTCTCGTATGC | 1 | (1104074) | 425 – 565 | KH36d, KH53b |
| val_p02_1 | pSMED02 | F: GGTGACAGATGGCCGAGAT  R: CTCGACGATCGGGCTCTTAC | 1 | (761420) | 403 – 467 | KH36d, KH53b |
| zwf | Chr | F: GGGGGCACCGGCGATCTTG  R: AGCGCAGTGCCATCAGATTCT | 1 | 770205 | 404 | KH12g, KH16b, KH46c |
